# A Comprehensive Survey on the Non-Invasive Passive Side-Channel Analysis

**DOI:** 10.3390/s22218096

**Published:** 2022-10-22

**Authors:** Petr Socha, Vojtěch Miškovský, Martin Novotný

**Affiliations:** Department of Digital Design, Faculty of Information Technology, Czech Technical University in Prague, 160 00 Prague, Czech Republic

**Keywords:** side-channel analysis, side-channel attacks, side-channel countermeasures, embedded systems, security

## Abstract

Side-channel analysis has become a widely recognized threat to the security of cryptographic implementations. Different side-channel attacks, as well as countermeasures, have been proposed in the literature. Such attacks pose a severe threat to both hardware and software cryptographic implementations, especially in the IoT environment where the attacker may easily gain physical access to a device, leaving it vulnerable to tampering. In this paper, we provide a comprehensive survey regarding the non-invasive passive side-channel analysis. We describe both non-profiled and profiled attacks, related security metrics, countermeasures against such attacks, and leakage-assessment methodologies, as available in the literature of more than twenty years of research.

## 1. Introduction

In the past few decades, computer systems and communication networks have become an essential part of our everyday lives. Various computing devices are used not only as tools for many professionals, but also for entertainment. These devices include embedded devices, such as payment cards, biometric passports, smart cars, trains, or whole cities, and even medical devices like pacemakers. Being surrounded by devices connected to the Internet, our private lives are endangered more than ever [1].

Special attention must therefore be given to ensure security of computer systems and their users. Various measures are employed to achieve confidentiality, integrity, availability, and non-repudiation of data with efficiency, ease of use, and cost in mind. Nowadays, widely used algorithms, such as Rijndael/AES [2,3] or RSA [4], are considered secure from the cryptoanalytic point of view. However, their implementations may leak sensitive information through the cryptographic device’s side channels, potentially compromising the entire system.

Side-channel attacks exploit the data-dependent side channels, such as power consumption of the cryptographic device [5,6] or its electromagnetic radiation [7], in order to extract secret information such as cipher keys. Such attacks pose a severe threat to both hardware and software cryptographic implementations, especially in the IoT environment where the attacker may easily gain physical access to a device, leaving it vulnerable to tampering. Various countermeasures have been proposed to prevent such attacks. Masking is a widely used technique based on randomization of the processed data [8,9,10,11], making it difficult to exploit the leakage. Hiding is another common approach, which aims to conceal the exploitable leakage in either side-channel signal amplitude or time [12,13,14,15]. Recent real-world attack examples show that uncompromising protection and testing of embedded cryptographic implementations is necessary [16].

This paper presents theoretical background and the state of the art in the area of non-invasive passive side-channel attacks. We map the history of this field and provide both a theoretical and practical overview. We present a systematic classification of both side-channel attacks and side-channel countermeasures and describe these. Therefore, our publication can serve as a good starting point for new side-channel researchers, as well as a universal reference. It is structured into seven sections as follows:

- Section 2, Side-Channel Security: Introduces side-channel leakage origin, measurement setup, formal model of the leakage, and leakage functions.
- Section 3, Non-Profiled Attacks: Describes non-invasive passive non-profiled attacks.
- Section 4, Profiled Attacks: Describes non-invasive passive profiled attacks.
- Section 5, Side-Channel Related Metrics: Describes both experimental and theoretical attack-related metrics.
- Section 6, Countermeasures Against Attacks: Describes hiding and masking countermeasures.
- Section 7, Attacks on Protected Implementations: Describes extensions of the presented attacks for attacking implementations with countermeasures.
- Section 8, Leakage Assessment: Describes methods for evaluations of side-channel leakage.

## 2. Side-Channel Security

Side channels of digital systems that may be used to compromise the system include power consumption [6,17,18,19], electromagnetic radiation [7], combinational logic delay [20,21], timing [5], and more. Some of these side channels are mutually dependent. For example, the relationship between current intensity and the magnetic field can be shown, e.g., by Biot–Savart law [22], and the combinational logic delay can be convincingly modeled as inversely proportional to the voltage drop [23]. This paper focuses on the dynamic power consumption side channel, but our presented concepts may be relevant for other side channels as well.

Side-channel attacks may be classified in many different ways [22], such as invasive/ non-invasive or active/passive. Invasive attacks require depackaging the chip in order to access internal components, such as data buses, whereas non-invasive attacks only exploit the external access. Active attacks tamper with proper functionality of the device (e.g., by introducing faults), whereas passive attacks only make use of observation of the device during its undisturbed operation. This paper focuses on non-invasive passive attacks only.

Side-channel attacks can also be classified as either horizontal or vertical. Horizontal attacks exploit leakage during a single algorithm execution, whereas vertical attacks exploit leakage from multiple executions. For example, considering a hardware implementation of the RSA algorithm that uses naïve square and multiply exponentiation, either only square operations are performed, or both square and multiply operations are performed during computation, for every exponent bit, depending on the bit being zero or one. This not only influences execution time, but, in some cases, it also allows the attacker to directly read the secret key from a single measured power/EM trace by graphing the trace, as the two operations form distinctive patterns [5]. This kind of a horizontal side-channel attack is called simple power analysis. Unlike this simple example, this paper focuses on vertical side-channel attacks, where the information is typically contained in the instantaneous signal amplitude as further described below.

**Figure 1:**
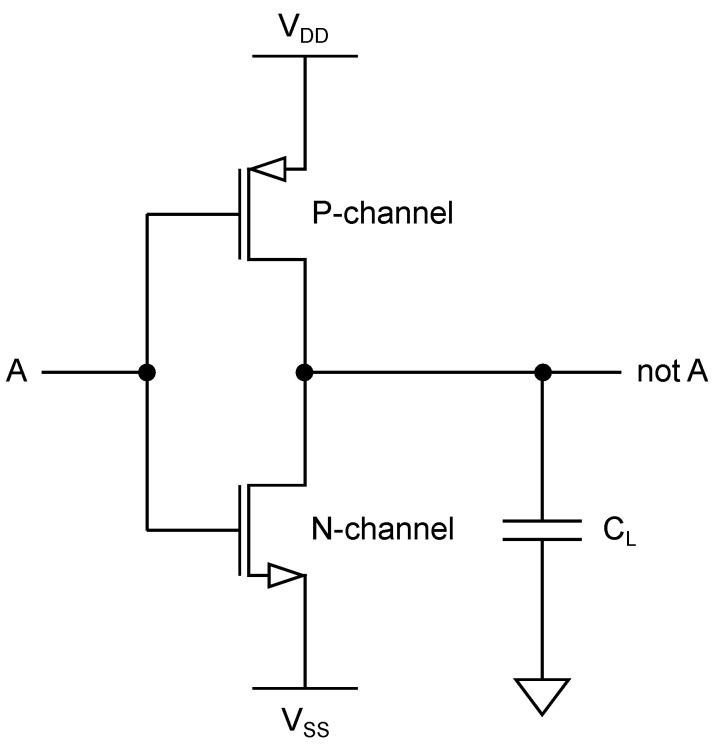
A CMOS inverter model.

A CMOS inverter model is depicted in Figure 1. Three different dissipation sources can be observed in such a CMOS structure [24]:

- static leakage current,
- short-circuit current, and
- capacitance charge and discharge.

**Figure 2:**
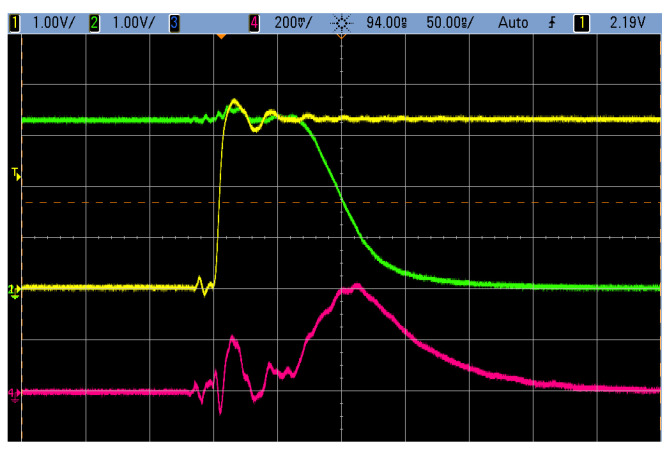
A CMOS inverter current consumption. The yellow line is the inverter input, the green line is the inverter output. The pink line is the current consumption, where peaks during the transition are clearly observable.

When the inverter input presents a stable voltage corresponding to 0 or 1, one of the transistors is open and the other one is closed. In this case, only static leakage current is present. When the input changes, short-circuit current can be observed for a brief period of time when both transistors are open. Furthermore, the modeled load capacitance $C_{L}$ has to be charged to the proper voltage when the input changes its value. Therefore, based on the instantaneous current consumption, it can be easily distinguished whether a transition happened or not. This fact is exploited by the most common leakage models as described later in this section. The consumption during a transition is demonstrated in Figure 2.

Because the P-channel MOSFET majority carriers have lower mobility and the minority carriers have lower lifetime, in contrast to the N-channel MOSFET [25,26], the P-channel MOSFET is typically built larger than the N-channel MOSFET [25], resulting in different characteristics, most importantly on-resistance and propagation delay (for non-inverter gates) [27]. Due to differences between N-channel and P-channel MOSFETs, the output value after transition can also be distinguished by the instantaneous current [28].

This simple example illustrates data dependency of the instantaneous power consumption, which is the main cause of the power-related side-channel information leakage in CMOS-based integrated circuits. Vertical attacks exploiting this kind of leakage typically require multiple side-channel measurements, unlike the previously described simple power analysis attack.

### 2.1. Measurements

**Figure 3:**
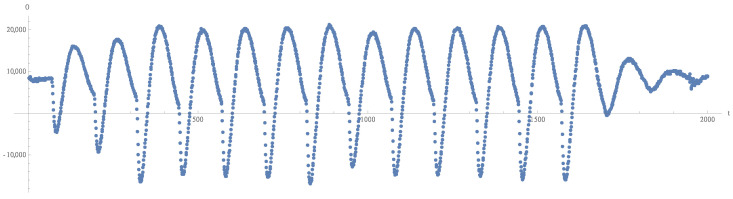
Rijndael/AES encryption FPGA power trace.

The device’s side channel is typically observed during a cryptographic operation, resulting in a measurement record, so-called trace, i.e., a vector of samples. For example, a single trace of dynamic power consumption during Rijndael/AES encryption in an FPGA is visualized in Figure 3. As mentioned earlier, multiple aligned traces, such as this one, are typically required for a successful attack, although single-trace attacks are sometimes also possible. This subsection briefly describes different measurement methods.

#### 2.1.1. Power Consumption

Power consumption of the cryptographic device is typically measured by using an oscilloscope which samples voltage across a shunt resistor. The current can then be obtained, knowing both resistance and voltage, by using Ohm’s law $I=\frac{U}{R}$. However, raw ADC values corresponding to the voltage can be directly used in a typical attack scenario, because the current and the voltage are assumed to be linearly dependent, as long as the oscilloscope setup parameters are consistent during all measurements.

Various measurement setups are described in [29], the differences being primarily in the shunt resistor placement.

**Figure 4:**
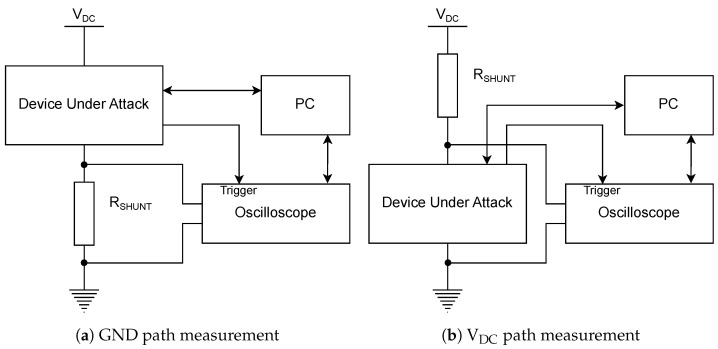
Example of a power measurement setup.

- Shunt resistor in GND path, with the voltage across the resistor being sampled by the oscilloscope, as shown in Figure 4a.
- Shunt resistor in V_DC_ path, with the voltage across the cryptographic device being sampled by the oscilloscope, as shown in Figure 4b, also observing voltage drops of the power regulator.

The latter setup offers an advantage when the device under attack has multiple power networks, because it allows the attacker to measure the just cryptographic core consumption. When the voltage is measured by using a shunt resistor in the ground path (Figure 4a), the measured voltage typically contains more noise such as noise caused by the device’s peripheral drivers. When measuring in the V_DC_ path (Figure 4b), the DC shift must be removed (unless measuring in a differential mode), which can be ensured either by using the oscilloscope’s AC mode, or by using external DC blocker. Other choices for power measurement include differential or current probes. However, these are not recommended unless necessary, as they present an additional source of environmental noise [29].

In a real-world attack, any decoupling capacitors near the cryptographic core must be removed, as they might filter out the relevant voltage changes. Correct power measurement setup is crucial for successful side-channel analysis. Parameters such as the environmental noise, sampling rate, or synchronization jitter have a direct impact on the attack success [30].

#### 2.1.2. Electromagnetic Radiation

Similarly to power measurement, an oscilloscope connected to a near-field probe may be used for measurement of electromagnetic radiation [7]. As mentioned earlier, there is a close relationship between the power consumption and the radiation [22].

Attacking electromagnetic radiation offers more degrees of freedom compared to the power analysis. The attacker can examine a particular part of the chip only, and she can choose from a wide variety of probes. Consequently, EM analysis may provide a very powerful tool at the cost of more intricate and more costly employment. Further discussion of EM side channels is outside of the scope of this paper.

In addition to directly measuring electromagnetic radiation of the device under attack, data-dependent leakage may also be unintentionally broadcast by a radio transmitter present on the same chip (such as SoC bluetooth/WiFi transmitters with built-in encryption) [31]. In mixed-signal systems on chip, the leakage from the digital part of the chip couples through substrate to the high-frequency analog radio transmitter [32]. This class of attacks is called screaming channels and it allows the attacker to successfully reveal cipher keys from traces obtained by a radio receiver from even 15 m distance [33].

#### 2.1.3. Combinational Logic Delay

**Figure 5:**
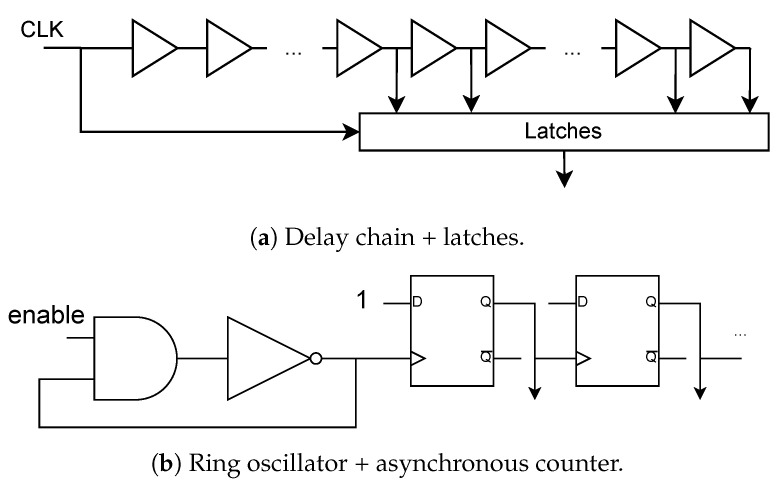
Combinatorial logic delay monitors.

Combinational logic delay inside a chip can be satisfactorily modeled as inversely proportional to the voltage drop of the internal power network [23], which is data-dependent due to the switching activity. In FPGA chips, the delay can be measured internally by using a delay-chain monitor [20,34], shown in Figure 5a, or by using a ring oscillator monitor [21], shown in Figure 5b. Acquired delay traces can be used in side-channel analysis in a similar fashion as the power traces.

Furthermore, crosstalk between two long wires inside the chip can be detected [35] by using a ring oscillator monitor as a receiver. In a multitenant FPGA chip setting, where independent customers share the same FPGA accelerator, e.g., in a cloud environment, these monitors open the possibility for remote and even automated large-scale side-channel attacks.

### 2.2. Formal Model

This subsection presents a formal model of side-channel leakage and corresponding terminology as described in [36,37]. The presented model is used for attack descriptions in the following sections.

Consider a physical device performing a cryptographic operation $E_{k}\left(x\right)$, depending on a secret (sub)key k∈K, where $K=B^{m}={\left\{0,1\right\}}^{m}$, x∈X. The unknown (sub)key is then modeled as a random variable K:Ω→K, the processed data as a random variable X:Ω→X.

Key-dependent state transitions (bit flips) occur inside the device during the execution of $E_{k}$. These state transitions are described as word pairs $(v_{1},v_{2})\in W$, where $W=B^{n}\times B^{n}$, $v_{1}$ is the previous state, and $v_{2}$ is the new state. Unknown transitions (word pairs) are modeled as a random variable W:Ω→W.

State transitions W induce side-channel leakage L on space L, modeled by a side-channel leakage function L(W). Leakage L is measured through the noisy physical observable O on space O.

**Figure 6:**
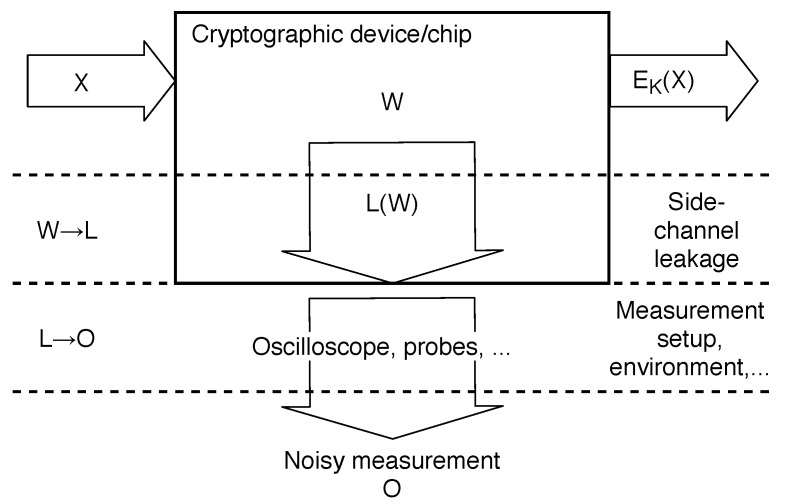
Illustration of channels involved in side-channel analysis.

The model describes a cascade of two channels W→L→O. This cascade is comprised of a leakage channel W→L through which information on processed words W leaks in L, and observation channel L→O through which the attacker obtains noisy information on L. The described channels are illustrated in Figure 6.

Observing O then means measuring $q\in N^{+}$ traces $o_{x_{i}}\left(t\right)$, i=1,2,…,q, of device’s side channel (e.g., power consumption) O(t), while processing known data $x_{i}$. In the case of Rijndael/AES, $o_{x_{i}}$ might be a trace similar to the one in Figure 3, and $x_{i}$ might be a corresponding plaintext or ciphertext block.

A side-channel attack is then defined as determining the (sub)key *k* by reconstructing the words W and using information on L contained in O. For example, the attack may be performed in these steps:

1. The real leakage function L is unknown, so the attacker assumes a hypothetical leakage function $\hat{L}$ (described in Section 2.3).
2. The attacker makes a guess $\hat{k}\in K$ on the real (sub)key *k*.
3. Based on the known data X, she computes an intermediate value $f_{\hat{k}}\left(X\right)$ within the $E_{k}$ computation.
4. The intermediate value implies a guess $W_{\hat{k}}$, which in turn implies a guess ${\hat{L}}_{\hat{k}}=\hat{L}\left(W_{\hat{k}}\right)$.
5. Finally, the attacker checks if the guess ${\hat{L}}_{\hat{k}}$ is compatible with the observed O.

This attack scenario assumes that the real (sub)key *k* is fully enumerable in a reasonable time and space. As shown in Section 3 and Section 4, side-channel attacks typically target a single subkey, e.g., an octet in the case of Rijndael/AES.

### 2.3. Leakage Function

Side-channel attacks can be classified into two groups according to the approach of the hypothetical leakage function $\hat{L}$:

- Non-profiled attacks, in which the attacker only makes use of an explicit leakage function, which is effective for a range of devices (e.g., based on CMOS technology) instead of being tailored for a specific one.
- Profiled attacks, which consist of a profiling step, in which the attacker examines a duplicate of the device under attack and she creates her own leakage approximation. Furthermore, her approximation inherently takes noise contained in O into account, making her empirical model more effective. This model is used for the attack, and an explicit leakage function may or may not be used during the process.

In addition to the differences regarding the approach to the hypothetical leakage function, these two types of attacks also assume a differently powerful attacker: for a profiled attack, an exact duplicate of the device under attack is required, whereas it is not for a non-profiled attack.

This subsection briefly introduces widely used explicit leakage functions necessary for non-profiled attacks, which are discussed thereafter in Section 3. Profiled attacks are discussed later in Section 4.

#### 2.3.1. Hamming Distance and Hamming Weight

A Hamming distance leakage function for $v_{1},v_{2}\in B^{n}$ is defined as a number of bit positions at which the words $v_{1},v_{2}$ differ [18]:

(1) $${\hat{L}}^{\mathrm{HD}}\left(v_{1},v_{2}\right)=\mathrm{HD}\left(v_{1},v_{2}\right)\in L=\left\{0,1,\ldots ,n\right\}.$$

The function corresponds to the number of bit flips in an *n*-bit wide register during $\left(v_{1},v_{2}\right)$ transition. It is a generally applicable model suitable for attacking CMOS logic. The Hamming distance is equal to the Hamming weight of XOR of the operands: $\mathrm{HD}\left(v_{1},v_{2}\right)=\mathrm{HW}\left(v_{1}⊕v_{2}\right)$, where Hamming weight HW is defined as a number of bits in the words that are set to one.

When $v_{1}$ is a zero vector, the Hamming distance leakage function reduces to Hamming weight leakage function [38],

(2) $${\hat{L}}^{\mathrm{HW}}\left(v_{2}\right)=\mathrm{HW}\left(v_{2}\right)\in L=\left\{0,1,\ldots ,n\right\},$$

for $v_{2}\in B^{n}$. This is often the case when attacking software implementations in microcontrollers [6,39].

Assuming Hamming weight $\mathrm{HW}\left(x\right),x\in B^{n}$ and uniformly distributed values of *n* bits in a word $x\in B^{n}$, the following properties hold for Hamming weight (and consequently Hamming distance),

(3) $$\mathrm{HW}\left(x\right)∼\mathrm{Binom}\left(n,\frac{1}{2}\right),$$

(4) $$E\left(\mathrm{HW}\left(x\right)\right)=\frac{n}{2},\;\;\mathrm{Var}\left(\mathrm{HW}\left(x\right)\right)=\frac{n}{4}.$$

Furthermore, the binomial distribution can be satisfactorily approximated by the normal distribution for $p=\frac{1}{2}$ [40], and therefore

(5) $$\mathrm{HW}\left(x\right)\approx N\left(\frac{n}{2},\sqrt{\frac{n}{4}}\right).$$

Generalized Distance Model

Less commonly, different weights may be assigned for 0→1 and 1→0 transitions, resulting in a generalized distance leakage function. For example, weight 1.5 may be used instead of 1 for the 1→0 transition to provide a more effective attack on some platforms [41].

#### 2.3.2. Identity

The identity leakage function [36]

(6) $${\hat{L}}^{\mathrm{id}}\left(f_{\hat{k}}\left(x_{i}\right)\right)=f_{\hat{k}}\left(x_{i}\right)\in L,$$

for $x_{i}\in X$, is equal to the targeted intermediate value within the $E_{k}\left(x_{i}\right)$ computation. It is the most general leakage function in the sense that it puts no assumptions on the cryptographic device or technology.

## 3. Non-Profiled Attacks

Non-profiled attacks can be divided into:

- parametric/moment-based attacks, which exploit statistical moments (such as mean or variance). Typical examples include differential power analysis [6] or correlation power analysis [17,18];
- non-parametric/information-theoretic attacks, which exploit the entire underlying statistical distribution. A typical example is mutual information analysis [36]; and
- machine learning-based attacks, namely the deep learning power analysis [42].

These attacks are presented in more depth in this section.

Unless stated otherwise, all of the attack descriptions in this section assume the attacker has already acquired $q\in N^{+}$ traces $o_{x_{i}}\left(t\right)$, i=1,2,…,q, of the device’s side channel O(t) (e.g., power consumption), while processing known data $x_{i}$ (e.g., plaintext), where bits in $x_{i}$ are uniformly distributed. Usage of uniform plain text gives a good confidence about uniformity of intermediate values during the computation, because a cipher where properties such as diffusion are expected is typically targeted.

The *q* measured traces can be modeled as *q* samples from a multivariate random variable O(t), where the dimension of the variable corresponds to a number of sampling points within single trace. All the attacks presented in this section, except for the last one, are univariate, i.e., only a single point in time is examined, which is desirable when the sensitive intermediate value manifests itself at a single time instant. In this section, unless stated otherwise, it is assumed that the interesting time instant t=τ is known and only the single relevant sampling point is considered. The *q* measured traces are therefore considered a univariate random variable O(τ).

In practice, when the time instant is unknown, the attack is performed at every time instant *t* independently. The final attack evaluation thus typically requires more attention and skill due to a larger false-result chance. Alignment of the traces is required when synchronization of the measurements (e.g., by using a trigger signal) is not possible.

### 3.1. Differential Power Analysis (DPA)

The differential power analysis [6] attack is performed in these steps:

1. Assume a single bit (n=1) Hamming weight (or distance) leakage function $\hat{L}$.
2. Enumerate (sub)key guesses $\hat{k}\in K$.
3. Compute an intermediate value $v_{2}=f_{\hat{k}}\left(x_{i}\right),\forall \hat{k},x_{i}$ (and the previous state $v_{1}$ if Hamming distance is used) and consider only a single bit (e.g., the LSB).
4. For every guess $\hat{k}$, partition measurements $o_{x_{i}}$ into two groups $O_{0}^{\hat{k}},O_{1}^{\hat{k}}$ according to the leakage function $\hat{L}$:
  (7) $$O_{0}^{\hat{k}}=\left\{o_{x_{i}}\;|\;\hat{L}\left(v_{1},f_{\hat{k}}\left(x_{i}\right)\right)=0\right\},$$
  (8) $$O_{1}^{\hat{k}}=\left\{o_{x_{i}}\;|\;\hat{L}\left(v_{1},f_{\hat{k}}\left(x_{i}\right)\right)=1\right\}.$$
5. Select the guess $\hat{k}$ for which the groups’ $O_{0}^{\hat{k}},O_{1}^{\hat{k}}$ means differ the most.
  - For wrong guesses $\hat{k}$, the traces for which L=0 and the traces for which L=1 are theoretically uniformly distributed in both groups.
  - For the right guess $\hat{k}$, the groups $O_{0}^{\hat{k}},O_{1}^{\hat{k}}$ should be distinguishable by their mean value, due to the bias caused by the fixed bit.

In the last step, the original Kocher’s DPA [6] selects the guess $\hat{k}$ for which the absolute difference of means between the two groups is greatest. More formally, the hypothesis about equal means may be examined by using Welch’s *t*-test or a similar statistic.

Example: Attacking First Round of Rijndael/AES

1. Assume Hamming weight or identity single-bit leakage (Hamming weight is equivalent to identity for n=1).
2. Select an enumerable key-dependent intermediate value during Rijndael/AES encryption: $f_{\hat{k}}\left(x_{i}\right):=\mathrm{Sbox}\left(x_{i}⊕\hat{k}\right)$. Sbox is an 8-bit bijection, $f_{\hat{k}}\left(x_{i}\right)$ is therefore computable for each byte independently of the other bytes.
3. Enumerate byte subkey guesses $\hat{k}\in K=\left\{0,1,\ldots ,255\right\}$, compute the intermediate value $v_{2}=f_{\hat{k}}\left(x_{i}\right),\forall \hat{k},x_{i}$ and choose, e.g., the LSB.
4. Use the Hamming weight of the LSB as leakage function and partition traces $o_{x_{i}}$ to groups $O_{0}^{\hat{k}},O_{1}^{\hat{k}}$.
5. Select the subkey guess $\hat{k}$ for which the two groups differ the most.

Because DPA builds its hypothesis on a single bit value, its assumptions regarding the leakage are very general. This may be one of the reasons for false results, so-called “ghost peaks”. The choice of the bit in $f_{\hat{k}}\left(x_{i}\right)$ has direct impact on the attack success. These facts are a motivation for multi-bit DPA as described further [43,44].

### 3.2. Multi-Bit DPA and Partitioning Power Analysis (PPA)

Bevan’s approach to multi-bit extension of the DPA is performing the original DPA independently for different bits of intermediate value $f_{\hat{k}}\left(x_{i}\right)$ and summing all the independent differences of means [43]. Then the subkey guess with the greatest summed difference is selected. This approach reduces the number of traces necessary as well as the chance of a false result [43].

Messerges’s multi-bit extension of the original DPA suggests using *n*-bit Hamming weight or Hamming distance leakage model [38], therefore utilizing the whole $f_{\hat{k}}\left(x_{i}\right)$ value. This time, two sets $O_{<}^{\hat{k}}$, $O_{\geq }^{\hat{k}}$ are defined so that

(9) $$O_{<}^{\hat{k}}=\left\{o_{x_{i}}\;|\;\hat{L}\left(f_{\hat{k}}\left(x_{i}\right)\right)<\frac{n}{2}\right\},$$

(10) $$O_{\geq }^{\hat{k}}=\left\{o_{x_{i}}\;|\;\hat{L}\left(f_{\hat{k}}\left(x_{i}\right)\right)\geq \frac{n}{2}\right\},$$

and their difference is examined similarly to the original DPA.

Partitioning power analysis [45,46] is a generalization of the multi-bit DPA. Assuming an *n*-bit $f_{\hat{k}}\left(x_{i}\right)$ intermediate value and a Hamming weight or Hamming distance leakage function, the traces $o_{x_{i}}$ are partitioned into (n+1) sets $O_{0}^{\hat{k}},\ldots O_{n}^{\hat{k}}$ so that

(11) $$O_{j}^{\hat{k}}=\left\{o_{x_{i}}\;|\;\hat{L}\left(f_{\hat{k}}\left(x_{i}\right)\right)=j\right\}.$$

The distinguishing statistic (which is a difference of means in the original DPA) is then defined by using weights $a_{j}\in R$ as

(12) $$\sum_{j=0}^{n}a_{j}\cdot \mu_{O_{j}^{\hat{k}}},$$

where $\mu_{O_{j}^{\hat{k}}}$ are means of the aforementioned groups.

The original DPA is a special case of 1-bit PPA where $a_{0}=-1,a_{1}=1$. Bevan’s 4-bit DPA is a special case of 4-bit PPA where $a_{0}=-\frac{1}{8},a_{1}=-\frac{1}{4},a_{2}=0,a_{3}=\frac{1}{4},a_{4}=\frac{1}{8}$. Messerges’s n-bit DPA is a special case of n-bit PPA where $a_{j}=-1$ for $0\leq j<\frac{n}{2}$, and $a_{j}=1$ for $\frac{n}{2}\leq j\leq n$ [46].

### 3.3. Correlation Power Analysis (CPA)

The correlation power analysis [17,18] attack is performed in these steps:

1. Assume a Hamming weight or Hamming distance leakage function $\hat{L}$.
2. Enumerate (sub)key guesses $\hat{k}\in K$.
3. Compute an intermediate value $v_{2}=f_{\hat{k}}\left(x_{i}\right),\forall \hat{k},x_{i}$ (and the previous state $v_{1}$ if using Hamming distance).
4. For every key guess $\hat{k}$, pairs $\left(o_{x_{i}},\;\hat{L}\left(v_{1},f_{\hat{k}}\left(x_{i}\right)\right)\right)$ represent samples from joint distribution $\left(O,{\hat{L}}_{\hat{k}}\right)$. (In other words, every trace is paired with the predicted Hamming weight/distance).
5. Compute Pearson correlation coefficient $\rho_{\hat{k}}=\frac{\mathrm{Cov}(O,{\hat{L}}_{\hat{k}})}{\sigma_{O}\sigma_{{\hat{L}}_{\hat{k}}}}$ for every $\hat{k}$.
6. Select the guess $\hat{k}$ for which the value of $|\rho_{\hat{k}}|$ is the highest.

Assuming there is a linear dependence between the predicted leakage and the physical observation, a significant correlation $\rho_{\hat{k}}$ should appear for the right guess $\hat{k}$, while for a wrong guess, the $\rho_{\hat{k}}$ should converge to zero.

Example: Attacking Last Round of Rijndael/AES

**Figure 7:**
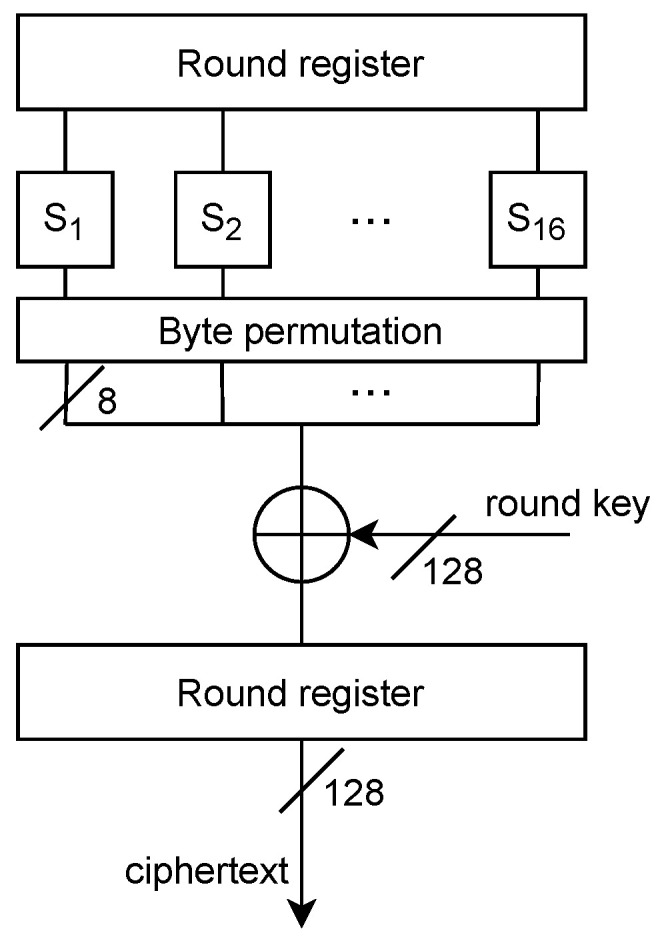
Architecture of Rijndael/AES last round. The scheme is unrolled for illustration purposes only; both “round register” blocks depict the same hardware register.

1. Assume the architecture illustrated in Figure 7 and a Hamming distance leakage (both $v_{1},v_{2}$ must be derived). Let $Y=E_{k}\left(X\right)$, i.e., ciphertext.
2. Let $v_{2}=y_{i}$. The previous register state is then $v_{1}=f_{\hat{k}}\left(y_{i}\right)={\mathrm{Sbox}}^{-1}\left({\mathrm{Perm}}^{-1}\left(y_{i}⊕\hat{k}\right)\right)$. Both values are once again enumerable for each byte independently of the other bytes due to the fact that the MixColumns operation is not performed in the last round.
3. Enumerate byte subkey guesses $\hat{k}\in K=\left\{0,1,\ldots ,255\right\}$, and compute the intermediate value $f_{\hat{k}}\left(y_{i}\right),\forall \hat{k},y_{i}$.
4. Compute the leakage function ${\hat{L}}^{\mathrm{HD}}\left(f_{\hat{k}}\left(y_{i}\right),y_{i}\right),\forall \hat{k},y_{i}$.
5. Compute Pearson correlation coefficient $\rho_{\hat{k}}=\frac{\mathrm{Cov}(O,{\hat{L}}_{\hat{k}})}{\sigma_{O}\sigma_{{\hat{L}}_{\hat{k}}}}$ for every $\hat{k}$.
6. Select the guess $\hat{k}$ for which the value of $|\rho_{\hat{k}}|$ is the highest.

Unlike previously described DPA attacks, which use a partitioning approach, the CPA attack uses a comparative approach. However, CPA with a single-bit leakage function is equivalent to the original DPA. Interestingly, CPA is equivalent to normalized PPA with weights implicitly given by the distribution of bits in $f_{\hat{k}}\left(x_{i}\right)$. For a uniform distribution of bits in $f_{\hat{k}}\left(x_{i}\right)$, CPA is equivalent to the Bevan’s multi-bit DPA [46].

The CPA attack assumes a linear relationship between the predicted leakage and the physical observation. However, this requirement can be relaxed to monotonicity by using the Spearman coefficient instead of the Pearson coefficient [47]. Similar to DPA, CPA exploits statistical moments such as mean or covariance, and therefore requires a “normally” distributed observation channel. The success of the attack largely depends on the quality of the leakage approximation and present noise.

### 3.4. Mutual Information Analysis (MIA)

The mutual information analysis [36] attack is performed in these steps:

1. Assume an arbitrary leakage function $\hat{L}\in L$ (with some restrictions, as explained in Section 3.4.1).
2. Enumerate (sub)key guesses $\hat{k}\in K$.
3. Compute an intermediate value $v_{2}=f_{\hat{k}}\left(x_{i}\right),\forall \hat{k},x_{i}$ (and the previous state $v_{1}$ if using Hamming distance).
4. Let $L_{0},\ldots ,L_{l}$ be subsets of L so that the set $\left\{L_{0},\ldots ,L_{l}\right\}$ is a partitioning of L. The elements $L_{j},j=0,\ldots ,l,$ are called atoms.
5. Associate inputs $x_{i}$ that leak $L_{j}$ under key guess $\hat{k}$ to $L_{j}^{\hat{k}}$:
  (13) $$L_{j}^{\hat{k}}=\left\{x_{i}\;|\;\hat{L}\left(v_{1},f_{\hat{k}}\left(x_{i}\right)\right)\in L_{j}\right\}.$$
  Each partition $\left\{L_{0}^{\hat{k}},\ldots ,L_{l}^{\hat{k}}\right\}$ induces a subdivision of measurements $o_{x_{i}}$.
6. Define conditional distributions ${\left\{P_{O\;|\;L_{j}^{\hat{k}}}\right\}}_{j=0}^{l}$ by using the subdivision of O, and let $P_{O}$, $P_{L_{\hat{k}}}$ be probability distributions of O, ${\hat{L}}_{\hat{k}}$.
7. Select $\hat{k}$ with the highest mutual information $I({\hat{L}}_{\hat{k}};O)$.

Unlike DPA or CPA, the mutual information analysis exploits mutual information, which is defined as

(14) $$I\left(X;Y\right)=D_{KL}(P_{X,Y}\;||\;P_{X}⊗P_{Y}),$$

where $D_{KL}$ is Kullback–Leibler divergence, i.e., a statistical distance describing probability distribution difference. Mutual information is directly related to entropy H:

(15) I(X;Y)=H(X)−H(X|Y)=H(X)+H(Y)−H(X,Y)=I(Y;X),

where H(X|Y) is conditional entropy, H(X,Y) is joint entropy. Mutual information can be intuitively interpreted as the amount of information obtained about X by observing Y or, in other words, the reduction of uncertainty in X obtained by observing Y.

The mutual information computation may go as follows:

1. Using measurements $o_{x_{i}}$ belonging to $L_{j}^{\hat{k}}$, estimate the conditional distribution $P_{O\;|\;L_{j}^{\hat{k}}}$ and the conditional entropy $\tilde{H}\left(O|{\hat{L}}_{\hat{k}}=j\right)$.
2. Compute the conditional entropy $\tilde{H}\left(O|{\hat{L}}_{\hat{k}}\right)$ by using ${\left\{\tilde{H}\left(O|{\hat{L}}_{\hat{k}}=j\right)\right\}}_{j=0}^{l}$.
3. By using all of the measurements $o_{x_{i}}$, estimate the distribution $P_{O}$ and the entropy $\tilde{H}\left(O\right)$.
4. Compute the mutual information $\tilde{I}\left({\hat{L}}_{\hat{k}};O\right)=\tilde{H}\left(O\right)-\tilde{H}\left(O|{\hat{L}}_{\hat{k}}\right)$.

Example: Attacking AES/Rijndael with Minimum Assumptions

1. Assume an identity leakage function $\hat{L}\left(f_{\hat{k}}\right)$, e.g., three MSBs of $f_{\hat{k}}\left(x_{i}\right)=\mathrm{Sbox}\left(x_{i}⊕\hat{k}\right)$.
2. Enumerate byte subkey guesses $\hat{k}\in K=\left\{0,1,\ldots ,255\right\}$, and compute the intermediate value $f_{\hat{k}}\left(x_{i}\right),\forall \hat{k},y_{i}$.
3. For every subkey guess, associate each $o_{x_{i}}$ with an atom of ${\left\{L_{i}^{\hat{k}}\right\}}_{i=0}^{7}$ based on its input’s predicted leakage.
4. For every subkey guess, estimate the densities $P_{O}$ and $P_{O|{\hat{L}}_{\hat{k}}}$ and compute the mutual information $\tilde{I}\left({\hat{L}}_{\hat{k}};O\right)$.
5. Select $\hat{k}$ with the highest mutual information $\tilde{I}\left({\hat{L}}_{\hat{k}};O\right)$.

Crucial aspect of mutual information analysis is the estimation of probability densities $P_{O}$ and $P_{O|{\hat{L}}_{\hat{k}}}$. Some of the choices include:

- histogram, i.e., a non-parametric discrete estimate;
- kernel density estimate, i.e., a non-parametric continuous estimate; and
- finite mixture model, i.e., a (semi-)parametric continuous estimate.

The quality of the estimate has a direct influence on both attack success and computational complexity [48,49].

A histogram provides a simple and efficient estimate with the most critical parameter being the number of bins [48]. The best estimate would require as many bins as there are values in the domain; however, it would be problematic to get enough values in every bin for it to be statistically significant. Less bins result in less information, but also lower susceptibility to noise. The original MIA [36] proposes using l+1 bins, i.e., as many bins as there are atoms in the L partitioning.

A kernel density estimate provides better attack results than the histogram [48] at the cost of its higher computational complexity. In this case, the kernel and bandwidth are the most critical parameters. Popular kernel choices include Epanechnikov (which is mean-square-error optimal) and Gaussian (for its convenience). Bandwidth has a similar role as bins in histograms, and it can be intuitively seen as a “smoothing parameter.” Generally speaking, the attacker aims to select the bandwidth as small as allowed by the data. A “rule-of-thumb” bandwidth estimator can be used alongside the Gaussian kernel [50].

A finite mixtures model assumes the underlying distribution to be a mixture of distributions, whose parameters are estimated, e.g., by using the expectation-maximization algorithm. Typically, a mixture of Gaussians is assumed [51].

Mutual information analysis puts no hard assumptions on the leakage function or the underlying distributions and provides sound results even with a simple identity leakage function. It provides a generic and powerful side-channel distinguisher (although it is less efficient in scenarios well-suited for DPA/CPA) [48,49,52].

#### 3.4.1. Leakage Function in Partitioning Attacks

Mutual information analysis allows for an arbitrary leakage function, giving the attacker a great degree of freedom. Although Hamming weight or Hamming distance leakage functions may be used when it is possible to predict both $v_{1},v_{2}$, their usage inherently leads to a loss of information. Identity leakage function, which is much more generic, may also be used. If the Hamming weight/distance estimate is possible, identity is shown to be less efficient, but still effective [48].

The leakage function must be selected so that a different $\hat{k}$ must not yield a permutation of ${\hat{L}}_{\hat{k}}$. For example, assume that using the identity of Rijndael/AES bijective S-box output. Different $\hat{k}$ then leads to a permutation of $\left\{L_{0}^{\hat{k}},\ldots ,L_{l}^{\hat{k}}\right\}$, which means that the mutual information is constant and independent of $\hat{k}$ [36]. This limitation can be easily overcome, e.g., by choosing only seven least significant bits of the Sbox output, or by using a Hamming weight/distance [36].

This problem does not only pertain to MIA, but to every partitioning attack (all the presented non-profiled attacks except CPA). When $f_{\hat{k}}\left(x_{i}\right)$ is an injective function, an attack using trivial partitioning where each value belongs in its distinct class will always fail [53,54].

### 3.5. Kolmogorov–Smirnov Analysis (KSA)

The Kolmogorov–Smirnov analysis [48,55] attack is performed in these steps:

1–6. The first six steps are same as for mutual information analysis in Section 3.4. Define the conditional distributions ${\left\{P_{O\;|\;L_{j}^{\hat{k}}}\right\}}_{j=0}^{l}$ and the distribution $P_{O}$.
7. For every $\hat{k}$, compute the average Kolmogorov–Smirnov distance between $P_{O}$ and $P_{O\;|\;L_{j}^{\hat{k}}}$, optionally further normalized by $\frac{1}{|O_{L_{j}^{\hat{k}}}|}$, where $|O_{L_{j}^{\hat{k}}}|$ is a size of the measurements set belonging to atom $L_{j}^{\hat{k}}$:
  (16) $$\underset{j}{E}(\frac{1}{|O_{L_{j}^{\hat{k}}}|}D_{KS}(P_{O}\;||\;P_{O\;|\;L_{j}^{\hat{k}}})).$$
8. Select the key guess $\hat{k}$ with the largest average KS-distance.

The Kolmogorov–Smirnov distance between $P_{X}$ and $P_{Y}$ is defined as

(17) $$D_{KS}(P_{X}\;||\;P_{Y})=\underset{x}{\sup}\left|F_{X}\left(x\right)-F_{Y}\left(x\right)\right|,$$

where $F_{X}$ is a cumulative density function of X. The Kolmogorov–Smirnov analysis is heavily inspired by the mutual information analysis. However, instead of estimating probability density function, the easier-to-obtain cumulative density function is used.

Alternatively, interclass Kolmogorov–Smirnov Analysis (iKSA) [56] distinguishes the key guess using the distance between the conditional distributions:

(18) $$\frac{1}{2}\underset{j,j^{\prime }}{E}(D_{KS}(P_{O\;|\;L_{j}^{\hat{k}}}\;||\;P_{O\;|\;L_{j^{\prime }}^{\hat{k}}})).$$

Other choices for comparison of the distributions include Cramér–von Mises criterion or different F-divergences [48].

The Kolmogorov–Smirnov Analysis shares some important characteristics with MIA, as both attacks can be used with an identity leakage function, and therefore without precise knowledge about the implementation and leakage. It can provide better results for weak signals than MIA due to its noise robustness [55].

### 3.6. Differential Deep Learning Analysis (DDLA)

The differential deep learning analysis [42] attack is performed in these steps:

1. Assume an arbitrary leakage function $\hat{L}\in L$.
2. Enumerate (sub)key guesses $\hat{k}\in K$.
3. Compute an intermediate value $v_{2}=f_{\hat{k}}\left(x_{i}\right),\forall \hat{k},x_{i}$ (and the previous state $v_{1}$ if using the Hamming distance).
4. Create labeled training datasets ${\left\{\left(o_{x_{i}},\hat{L}\left(v_{1},f_{\hat{k}}\left(x_{i}\right)\right)\right)\right\}}_{\hat{k}},\forall \hat{k},o_{x_{i}}$. Note that the same limitations as described in Section 3.4.1 apply.
5. Perform deep learning classifier training for every dataset.
6. Select the key guess $\hat{k}$ with the best DL training metrics.

Unlike the previously described attacks in this section, the differential deep learning analysis is typically used in a multivariate fashion, not univariate. In other words, the attack is not performed at a single sampling point or all the sampling points in the trace independently. Instead, the classifier is fed with the multivariate vectors corresponding to the entire encryption.

The differential deep learning analysis is a partitioning attack, like all the previously presented attacks except CPA. A key-dependent partitioning of the data is created and then the distinguishability of the partitions is examined by using the classifier. For the correct key guess, the classifier should be able to learn distinctive features of differently labeled data. When a wrong guess is made, the traces are randomly distributed across labels, and therefore the training metrics should be significantly worse than for the correct guess. Different training metrics are proposed for the final selection of the key, e.g., by using sensitivity analysis [42].

Various deep-learning architectures, such as a multilayer perceptron or a convolutional network may be used for the classifier. Translation-invariance property of convolutional networks can be exploited to attack desynchronized traces [42,57], whereas previously described attacks would require synchronization of the traces during preprocessing, e.g., by using autocorrelation, due to their univariate nature. A distinct disadvantage of using the machine-learning-based blackbox approach is limited explainability of the results [58].

## 4. Profiled Attacks

Profiled attacks assume the attacker has a fully controlled identical copy of the device under attack at her disposal. She is capable of observing the device’s side channels during execution of the identical cryptographic implementation. Moreover, she is able to feed the implementation with arbitrary inputs and keys. Her attack is tailored for a specific device and therefore more effective and efficient than a nonprofiled attack.

A profiled attack consists of two phases:

1. Profiling phase, during which an empirical model of the leakage is created by using the identical copy of the device under attack.
2. Attack phase, during which observations of the device under attack are evaluated by using the previously profiled model.

Unlike non-profiled attacks presented in Section 3, all the presented profiled attacks are multivariate, i.e., full traces O(t) are considered, where the dimension (*t*) corresponds to a number of sampling points within a single trace.

### 4.1. Template Attack (TA)

The template attack [19,59] is performed in these steps:

1. Consider an arbitrary leakage function $\hat{L}\in L$.
  Profiling phase
2. Measure a profiling set of traces $o_{\left(x_{i},k_{i}\right)}\left(t\right)$ using desired (typically, but not necessarily random uniform) inputs $\left(x_{i},k_{i}\right)$.
3. Compute an intermediate value $v_{2}=f_{k_{i}}\left(x_{i}\right),\forall k_{i},x_{i}$ (and the previous state $v_{1}$ if using Hamming distance).
4. Let $L_{0},\ldots ,L_{l}$ be subsets of L so that the set $\left\{L_{0},\ldots ,L_{l}\right\}$ is a partitioning of L. The elements $L_{j},j=0,\ldots ,l,$ are called atoms.
5. Associate measurements $o_{\left(x_{i},k_{i}\right)}$ whose inputs $\left(x_{i},k_{i}\right)$ leak $L_{j}$ to $O_{j}$:
  (19) $$O_{j}=\left\{o_{\left(x_{i},k_{i}\right)}\;|\;\hat{L}\left(v_{1},f_{k_{i}}\left(x_{i}\right)\right)\in L_{j}\right\}.$$
6. Select points of interest $t_{i}$ within the measurements $o_{\left(x_{i},k_{i}\right)}\left(t\right)$, e.g., by using sum of differences of average traces of each $O_{j}$ set or by using principal component analysis. From this moment on, restrict the measurements to these points only.
7. Create empirical models, so-called templates, $T_{j}$, e.g., Gaussian probability estimates, characterizing leakage induced by atoms $L_{j}$ using traces in set $O_{j}$.
  Attack phase
8. Measure an attack set of traces $o_{x_{i}}\left(t\right)$ using desired plain texts $x_{i}$.
9. Enumerate (sub)key guesses $\hat{k}\in K$.
10. Compute an intermediate value $v_{2}=f_{\hat{k}}\left(x_{i}\right),\forall \hat{k},x_{i}$ (and the previous state $v_{1}$ if using Hamming distance).
11. Associate measurements whose inputs $x_{i}$ leak $L_{j}$ under key guess $\hat{k}$ to $O_{j}^{\hat{k}}$:
  (20) $$O_{j}^{\hat{k}}=\left\{o_{x_{i}}\;|\;\hat{L}\left(v_{1},f_{\hat{k}}\left(x_{i}\right)\right)\in L_{j}\right\}.$$
12. Compute probabilities $Pr\left(O=o_{x_{i}}\;|\;L\in L_{j}\right),o_{x_{i}}\in O_{j}^{\hat{k}}$ that measurements in $O_{j}^{\hat{k}}$ leak $L_{j}$ using the templates $T_{j}$.
13. Select the key guess $\hat{k}$ with the highest overall probability (product of posterior probabilities) of the predicted leakage.

The template attack provides the attacker with a very powerful and universal tool. The empirical templates $T_{j}$ are typically multivariate Gaussian models [19]. A creation of the Gaussian template is demonstrated in the following examples.

Example: Attacking Rijndael/AES Using Hamming Weight and Gaussian Templates

1. Assume the Hamming weight leakage function $\hat{L}$ and intermediate value $f_{\hat{k}}\left(x_{i}\right)=\mathrm{Sbox}\left(x_{i}⊕\hat{k}\right)$.
  Profiling phase
2. Measure a profiling set of traces $o_{\left(x_{i},k_{i}\right)}\left(t\right)$, by using random uniform inputs $\left(x_{i},k_{i}\right)$.
3. Partition measurements $o_{\left(x_{i},k_{i}\right)}\left(t\right)$ into nine groups according to the Hamming weight of S-box output:
  (21) $$O_{j}=\left\{o_{\left(x_{i},k_{i}\right)}\left(t\right)\;|\;\hat{L}\left(f_{k_{i}}\left(x_{i}\right)\right)=j\right\}.$$
4. Select sampling points of interest $t_{1},\ldots ,t_{m}$ using sum of differences of average traces:
  (a) Compute the average measurement $M_{j}\left(t\right)$ for every group $O_{j}$.
  (b) Compute the sum of the absolute pairwise differences of these average power traces: $\sum_{i,j}\left|M_{i}\left(t\right)-M_{j}\left(t\right)\right|$ and select the most deviate points, preferably in different clock cycles.
  Reduce the dimensionality of O(t) and of the average traces $M_{j}\left(t\right)$ to these selected points only.
5. Define noise measurements as
  (22) $$N_{j}=\left\{n_{\left(x_{i},k_{i}\right)}\left(t\right)\;|\;n_{\left(x_{i},k_{i}\right)}\left(t\right)=o_{\left(x_{i},k_{i}\right)}\left(t\right)-M_{j}\left(t\right)\wedge o_{\left(x_{i},k_{i}\right)}\left(t\right)\in O_{j}\right\},$$
  and consider $N_{j}$ samples from variable $N_{j}$.
6. Compute the noise covariance matrices $\Sigma_{j}$ between all the points of the interest for every group $N_{j}$:
  (23) $$\Sigma_{j}=\left(\begin{array}{ccc} \mathrm{Var}(N_{j}\left(t_{1}\right)) & \cdots & \mathrm{Cov}(N_{j}\left(t_{1}\right),N_{j}\left(t_{m}\right))\\ \vdots & \ddots & \vdots \\ \mathrm{Cov}(N_{j}\left(t_{m}\right),N_{j}\left(t_{1}\right)) & \cdots & \mathrm{Var}(N_{j}\left(t_{m}\right)) \end{array}\right).$$
7. $T_{j}=\left(M_{j},\Sigma_{j}\right)$ is a Gaussian template characterizing leakage L=j, i.e., Hamming weight of the S-box output.
  Attack phase
8. Measure/capture an attack set of measurements $o_{x_{i}}\left(t\right)$, by using random uniform plaintexts $x_{i}$.
9. Enumerate byte subkey guesses $\hat{k}\in K=\left\{0,1,\ldots ,255\right\}$ and compute the intermediate value $v_{2}=f_{\hat{k}}\left(x_{i}\right),\forall \hat{k},x_{i}$.
10. Partition the traces $o_{x_{i}}\left(t\right)$ into nine groups according to the predicted Hamming weight, for every subkey guess:
  (24) $$O_{j}^{\hat{k}}=\left\{o_{x_{i}}\left(t\right)\;|\;\hat{L}\left(f_{\hat{k}}\left(x_{i}\right)\right)=j\right\}.$$
11. For every measurement $o_{x_{i}}\left(t\right)$ in group $O_{j}^{\hat{k}}$, evaluate the probability of it belonging in the designated group by evaluating the template $T_{j}=\left(M_{j},\Sigma_{j}\right)$:
  (a) Compute hypothetical noise vector $n_{x_{i}}\left(t\right)=o_{x_{i}}\left(t\right)-M_{j}\left(t\right)$.
  (b) Compute the probability of observing $n_{x_{i}}\left(t\right)$ by using the multivariate Gaussian probability distribution:
    (25) $$p_{j}\left(n_{x_{i}}\left(t\right)\right)=\frac{1}{\sqrt{{\left(2\pi \right)}^{N}\left|\Sigma_{j}\right|}}\exp\left(-\frac{1}{2}\;\;n_{x_{i}}{\left(t\right)}^{⊤}\;\;\Sigma_{j}^{-1}\;\;n_{x_{i}}\left(t\right)\;\right),$$
    where *N* is number of points of the interest, $|\Sigma_{j}|$ is the determinant of $\Sigma_{j}$, and $\Sigma_{j}^{-1}$ is its inversion.
12. Select the key guess $\hat{k}$ with maximum overall probability of the measurements being partitioned in the correct groups.

Example: Attacking Rijndael/AES Using a Single Measurement

1. Assume identity leakage function $\hat{L}$ and intermediate value $f_{\hat{k}}\left(x_{i}\right)=\hat{k}$. Assume an atom $L_{j}$ for every subkey value, i.e., 256 atoms.
  Profiling phase
2. Measure a large amount of traces by using uniform plain texts and keys and create 256 Gaussian templates.
  Attack phase
3. Measure a trace using a uniform plaintext and evaluate it against all the templates. Select the key guess with the highest probability.

Computing the probabilities as described above may lead to numerical instabilities, which can be solved by using logarithms of probabilities instead [28].

The creation of the model (i.e., the templates) requires a large amount of measurements in comparison to the actual attack. Efficient and effective templates may require further evaluation due to potential overfitting; templates too specific for the attacker’s copy might not work on the device under attack [19].

Various ways to improve the efficiency of the template attack are described in [60], such as methods for the dimensionality reduction/selection of points of the interest, usage of pooled covariance matrices, combining multiple traces, etc.

Many use cases and scenarios are possible by using the extend-and-prune approach, i.e., starting with small parts of information and increasingly extending the attack. Thanks to the profiling phase, the attack phase is very effective and efficient.

### 4.2. Machine Learning-Based Attacks

Machine learning (ML) algorithms are algorithms that learn to solve a problem without being explicitly programmed to do so. In this context, “to learn” can be perceived as “to build an empirical model using training data”, whereas “to solve” can be perceived as “to evaluate real data using the model”. A profiled side-channel attack as described in this section can be reduced to a classifying task, which is thoroughly studied in the context of supervised machine learning [61].

Profiled machine learning-based attacks are typically performed in a similar fashion as the template attack, and can often be classified as one. They also share many of its advantages and disadvantages. The crucial difference is in the choice of the empirical model; instead of Gaussian, a machine learning-based classifier is used.

A support vector machine (SVM) is a machine learning algorithm commonly used for classification, based on creating an optimal hyperplane between different classes. It was shown to be more efficient than a Gaussian template attack in some aspects [62,63,64], performing better on noisy measurements and requiring a smaller profiling set. However, selection of the algorithm parameters, such as the kernel function, may have a significant impact on its performance [62]. Both binary and multi-class SVM classifiers were successfully used to attack Rijndael’s S-box output [65]. Other common classifier choices are decision trees, random forests [64,66], and others.

Neural network-based deep learning classifiers are a popular choice in side-channel security [67,68]. The non-profiled variant of the attack is presented in Section 3.6. Both multilayer perceptron [69,70] and convolutional neural network [57,71] architectures are suitable for a profiling attack. Whereas a multilayer perceptron classifier must be fed with aligned power traces, the location and scale invariant convolutional neural network can extract the features itself and therefore it is capable of processing misaligned or jittered measurements without prior preprocessing [57]. It is capable of exploiting both univariate and multivariate leakage, as well as utilizing both Hamming weight/distance or identity training labels [72].

Hyperparameters of the neural network model include the network architecture (number of nodes in a layer, number of layers, activation function) and learning parameters (number of epochs, batch size, optimization algorithm, learning rate). Unfortunately, there does not seem to be the best model for every scenario (“no free lunch” theorem). Finding a suitable model is a nontrivial task; however, it is crucial for a successful attack. Class imbalance may present a significant obstacle [73], especially when Hamming weight is used (e.g., only a single word value $x\in B^{n}$ leads to HW=0, in contrast to $\mathrm{HW}=\frac{n}{2}$). Even though the neural network can be fed the whole unprocessed and even misaligned traces, it holds that the higher is the dimension of the data, the higher is the attack complexity and the larger training sets are required [66]. Similarly to the template attack, both underfitting and overfitting the of model during the learning phase may lead to an unsuccessful attack; the former cannot generalize the observations, whereas the latter learns non-relevant details and noise [72].

## 5. Side-Channel Attack-Related Metrics

Several metrics related to side-channel attacks are presented in this section. Experimental metric success rate and Guessing entropy are presented in Section 5.1. Theoretical metrics’ confusion coefficients and distinguishing margins, and their relationship to differential cryptoanalysis are presented in Section 5.2.

Let $\hat{k}\in K$ be a (sub)key guess during an attack and let $k^{*}\in K$ be the real (secret) (sub)key. Define a distinguisher $D^{\hat{k}}\left(o_{x_{1}},\ldots ,o_{x_{q}};x_{1},\ldots ,x_{q}\right)$ as an absolute value of the statistic that is used to distinguish the correct key during the attack. For example, a difference of means or t-value in case of DPA (Section 3.1), a correlation coefficient in case of CPA (Section 3.3), a mutual information in case of MIA (Section 3.4), a Kolmogorov–Smirnov distance in case of KSA (Section 3.5), probabilities in case of DDLA (Section 3.6), and TA (Section 4.1). Assume that the higher the value of $D_{\hat{k}}$ is, the higher is the probability of the correct key $\hat{k}$.

### 5.1. Success Rate and Guessing Entropy

Success rate [74] and guessing entropy [75,76] are experimental metrics allowing a comparison of different attacks on the same implementation. A simplified definition of these metrics is presented in this subsection, omitting two parameters: time complexity τ and memory complexity *m* [74].

Assume all the key guesses $\hat{k}$ are sorted according to the value of $D^{\hat{k}}$ in descending order, i.e., the most probable candidate is positioned first, the second most probable candidate is positioned second, etc. Let $\#(\hat{k})$ be a position of the guess $\hat{k}$. Success rate is then defined as the probability of the correct key guess $k^{*}$ being on the first position:

(26) $$\mathrm{SR}\left(q\right)=\mathrm{Pr}(\#\left(k^{*}\right)=1),$$

where *q* is the number of measurements available. In other words, it is the probability of the attack revealing the correct key. The *n*-th order Success rate is defined as

(27) $${\mathrm{Succ}}^{n}\left(q\right)=\mathrm{Pr}(\#\left(k^{*}\right)\leq n).$$

Guessing entropy is a related metric defined as the expected position of the correct key within the previously mentioned sorted guesses:

(28) $$\mathrm{GE}\left(q\right)=E(\#\left(k^{*}\right)),$$

where *q* is a number of measurements available. Whereas success rate characterizes the probability of the attack being successful, guessing entropy characterizes the amount of the remaining work of the attacker when the attack fails to reveal the correct key.

In order for the values of success rate or guessing entropy to be trustworthy, a large number of independent experiments must be performed [74].

### 5.2. Confusion Coefficient and Distinguishing Margin

Consider a single-bit intermediate value $f_{k}\left(x\right)$ (as in a DPA attack). Let $k^{*}$ be the correct key and k∈K be any key hypothesis. The confusion coefficient $\kappa (k^{*},k)$ is then defined as a probability of the bit $f_{k}\left(x\right)$ having a different value given two different keys [77]:

(29) $$\kappa \left(k^{*},k\right)=\mathrm{Pr}\left(f_{k^{*}}\left(x\right)\neq f_{k}\left(x\right)\right).$$

It reaches minimum when the two keys are the same: κ(k,k)=0, and maximum $\kappa (k^{*},k)=1$ iff $\exists k\neq k^{*},\forall x:f_{k^{*}}\left(x\right)=\bar{f_{k}\left(x\right)}$. Assuming $f_{k}\left(x\right)=S\left(x⊕k\right)$, the confusion coefficient is directly linked to cryptoanalytical metrics of the boolean S-box $S:B^{n}\to B^{m}$, namely to its differential uniformity $\Delta_{S}$:

(30) $$\Delta_{S}=\max_{a\in B^{m},k\in B^{n}}\left|\left\{x\in B^{n}\;|\;S\left(x\right)⊕S\left(x⊕k\right)=a\right\}\right|.$$

Considering m=1 ($f:B^{n}\to B^{m}$ being equivalent to ${\left\{f_{i}:B^{n}\to B\right\}}_{i=1}^{m}$) [78]:

(31) $$2^{-n}\Delta_{S}-\frac{1}{2}=\max_{k\neq k^{*}}\;\left|\kappa \left(k^{*},k\right)-\frac{1}{2}\right|.$$

Let *b* be one bit of a sensitive variable $f_{k}\left(x\right)$ for a perfectly secret encryption algorithm. Then *b* is equiprobable, i.e., $\mathrm{Pr}\left(b=1\right)=\mathrm{Pr}\left(b=0\right)=\frac{1}{2}$ [79]. Assume that the bit *b* is the one under attack. In such a case, the DPA/CPA and KSA/iKSA distinguishers can be rewritten in following closed-form expressions [78]:

(32) $$D_{\mathrm{CPA}}^{k}=D_{\mathrm{DPA}}^{k}=\frac{2}{\sqrt{1+1/\mathrm{SNR}}}\cdot \left|\kappa \left(k^{*},k\right)-\frac{1}{2}\right|,$$

(33) $$D_{\mathrm{KSA}}^{k}=2D_{\mathrm{iKSA}}^{k}=\left(2\Phi \left(\sqrt{\mathrm{SNR}}\right)-1\right)\cdot \left|\kappa \left(k^{*},k\right)-\frac{1}{2}\right|,$$

where Φ(x) is a cumulative distribution function of the standard noise N(0,1). These equations describe the relationship between the distinguisher and noise. Notice that for large noise, the first multiplicand in both equations tends to zero.

Let the distinguishing margin (distance to the nearest rival) be the distance between the correct key $k^{*}$ distinguisher value $D^{k^{*}}$ and the maximum incorrect key guess $\hat{k}$ distinguisher value [80]:

(34) $$\mathrm{DM}=D^{k^{*}}-\max\left\{D^{\hat{k}}|\hat{k}\neq k^{*}\right\}.$$

The distinguishing margin characterizes the ability of the attacker to distinguish the correct key, i.e., her ability to make the attack succeed. For the Kolmogorov–Smirnov distinguisher, it can be explicitly expressed in terms of confusion coefficient, and therefore differential uniformity [78]:

(35) $${\mathrm{DM}}_{\mathrm{KSA}}=\lambda \;(\frac{1}{2}-\max_{k\neq k^{*}}\;|\kappa \left(k^{*},k\right)-\frac{1}{2}\left|\right)=\lambda \left(1-2^{-n}\Delta_{S}\right).$$

This equation demonstrates that the attack becomes easier as the distance between κ and $\frac{1}{2}$ becomes smaller. It also provides a direct link between S-box properties, i.e., its differential uniformity, and its susceptibility to side-channel attacks; the harder the differential cryptoanalysis, the easier the side-channel analysis.

## 6. Countermeasures against Attacks

Countermeasures against side-channel attacks can be categorized into two basic groups [28]:

- Hiding, whose main objective is to “hide” the sensitive variable leakage, ideally to entirely remove the data dependency of the L→O channel. Hiding countermeasures generally focuses on the signal-to-noise ratio (recall Equations (32) and (33)). Hiding countermeasures can sometimes be further classified as (1) hiding in amplitude, and (2) hiding in time. Shuffling, which randomizes the algorithm flow, is sometimes considered a separate category, as it is implemented on the algorithm level; however, its effect is similar to that of hiding.
- Masking randomizes the processed data W while still providing correct results, therefore making it hard (ideally impossible) for the attacker to predict any intermediate values. The aim is to make the W→L channel appear random, ideally to remove the data dependency altogether. Unlike hiding countermeasures, masking typically requires a source of fresh randomness. The security of the masking schemes is therefore dependent on the used random generator.

Correctly employing the presented countermeasures does not result in an absolutely secure implementation. The objective is to make the attack infeasible in a real-world scenario, typically by increasing either the number of measurements necessary or the computational cost of the attack over a limit of resources practically available to the attacker. Similar to the classic cryptoanalysis, this limit lowers in time as the available computational capacity increases. Real-world attack examples [16] in 2021 show that protection against extreme numbers of traces (hundreds of millions or more) is necessary. Attacks on protected implementations are presented later at Section 7.

In implementation terms, the countermeasures can further be categorized into three groups [81]:

- Secure logic styles, which incorporate custom logic gate libraries, designed to minimize the data dependency. These countermeasures inherently introduce a large overhead, and their implementation is typically expensive. Countermeasures in this category are generally “hiding in amplitude”.
- Additional modules, which are incorporated into the cryptographic design. The basic advantage of these modules is their universal applicability and lower cost compared to that of the secure logic style. Countermeasures in this category are generally hiding countermeasures, either in amplitude or in time.
- Cryptographic module modifications, which aim to alter the encryption itself. While offering a lower overhead than the previously mentioned categories, they are often algorithm-specific and may present unforeseen weaknesses. This category contains, most importantly, masking. However, some hiding countermeasures fall within the category as well.

Additionally, some types of cryptography were believed to be resistant to SCA by their nature. For example, chaos cryptography [82,83] was expected to be hard to attack because of its unpredictable behaviour [84]. Nevertheless, these beliefs were disproven, e.g., by a study showing that the chaos-based S-Boxes are similarly vulnerable to SCA as the AES ones [85]. Similarly, ARX-based cryptography [86,87] was expected to be more resistant to SCA, as there is no highly nonlinear element (S-Box) whereas later research showed the opposite [88,89].

### 6.1. Secure Logic Styles

The logic gates are typically designed in static CMOS technology, as illustrated in Figure 1). Various logic styles aim to compensate for their data-dependent behavior, often combining concepts of differential and dynamic logic [24]. Differential logic uses complementary signals (e.g., *A* and $\bar{A}$) at gate input and output, implementing differential pull-down networks to equalize the power consumption of different transitions. Two-phase dynamic logic introduces clock-driven pre-charge, where the load capacitance is artificially charged. Combination of the two styles is often referred to as dynamic differential logic or dual-rail pre-charge logic [81].

Various countermeasures based on secure logic styles were proposed in the literature. Sense amplifier-based logic (SABL) [12] is an example of dynamic differential logic. It aims to ensure constant consumption of all input and output transitions at the cost of complicated design (custom logic gates, “domino” logic), under the assumption that all interconnections and capacitances are symmetrical (balanced). Simple dynamic differential logic (SDDL) [13] uses ordinary CMOS gates, implementing the differential logic by using De Morgan’s law and the pre-charge using AND gates. Wave dynamic differential logic (WDDL) [13] extends the SDDL by limiting the used logic to AND and OR gates, thanks to which a “precharge wave” is introduced to reduce overhead. Furthermore, WDDL promises to be glitch-resistant, as opposed to SDDL, where data-dependent hazards may compromise the countermeasure. Similarly to sense amplifier-based logic, SDDL and WDDL require symmetrical interconnections and capacitances. Other extensions of dynamic differential logic are available in the literature [90,91,92].

Adiabatic logic [93] was originally designed for low-power applications with the aim of reusing energy efficiently instead of it being discharged. It is powered by a clock-controlled power source, typically trapezoidal. Various logic styles which make use of adiabatic logic were proposed with the aim of hiding leakage [94,95].

Other logic style examples include randomized multitopology logic [96] or use of asynchronous logic styles [97,98,99].

### 6.2. Additional Modules

Unlike countermeasures based on secure logic styles, the countermeasures presented in this subsection put no (or minor) assumptions on the cryptographic module.

Measured SNR can be effectively lowered by employing noise generators inside the cryptographic device. Different primitives can be utilized to build the generator, including shift registers, block RAMs, switch boxes [14] or ring oscillators [100]. The design of the actual cryptographic primitive can be used for correlated noise generation [101,102].

Current sense-shunt loop-back can be used for active current flattening to hide the leakage [103,104]. Similarly, the current can be randomized by using a variable current source [105]. Decoupling-based countermeasures are based on powering the cryptographic core from internal capacitors [106,107,108] in order to hide the instantaneous consumption.

Hiding in time is typically achieved by employing a specific clock signal or by altering the cryptographic algorithm. Isolated clock network can be used to deny the attacker the possibility of using the global clock network for synchronization [109]. The clock signal can be randomized [14]. Alternatively, dummy operations and data can be inserted randomly during the computation [110,111,112]. Partial dynamic reconfiguration can be used to shuffle the algorithm execution to hide the leakage in both time and amplitude [15].

### 6.3. Masking

Unlike previously presented countermeasures, masking [8] requires a detailed knowledge of the cryptographic algorithm. Its implementation is modified so that all intermediate values are masked by using a random value, making it difficult for an attacker to predict the leakage and therefore mount an attack. A relevant function (group law) is chosen for the masking according to the values domain, e.g., an exclusive or (XOR) in case of Galois field—then the masking is called boolean. When the sensitive value is multiplied by a mask, the masking is called multiplicative. Unlike boolean masking, multiplicative masking is inherently unable to mask a zero value [113].

Unless stated otherwise, boolean masking is considered in this subsection. The sensitive value *x* is split into d+1 shares $x_{i}$, where

(36) $$x=\overset{d}{\underset{i=0}{⨁}}x_{i}.$$

The splitting is done by generating *d* uniform random masks $x_{1},\ldots ,x_{d}$ and by putting $x_{0}=x⊕x_{1}⊕\ldots ⊕x_{d}$. The number of masks *d* is then called a masking order. Implementation secured with *d*-order masking should ideally be secure against attacks up to *d*-th order [8,114] (as defined further in Section 7). However, the desired security level is often not reached in practice [115,116] due to unforeseen imperfections.

A cryptographic algorithm typically consists of several linear and nonlinear operations, some of which must be altered to function properly when the variable is split. Masking of a linear operation *f* is trivial because all of the shares can be processed independently:

(37) $$f\left(x\right)=f\left(x_{0}⊕\ldots ⊕x_{d}\right)=f\left(x_{0}\right)⊕\ldots ⊕f\left(x_{d}\right).$$

There are different approaches to dealing with the nonlinear operations. Considering substitution-permutation network-based ciphers, substitution boxes (S-boxes) are typically the nonlinear operations.

Pre-computed masked S-boxes were originally proposed for first-order masked software implementations [9] and later adapted for hardware [14,117]. The concept is further described in Section 6.3.1.

A more efficient approach suitable for hardware Rijndael/AES implementations splits the S-box into an inversion and an affine operation and masks the inversion by using a multiplicative mask [118]. Even lower overhead can be achieved when the S-box computation is performed in a composite field [119,120,121]. However, these hardware masking schemes were later shown to be vulnerable against first-order side-channel attacks due to data-dependent glitches occuring during the S-box computation [115]. An example of glitch-induced leakage in a masked AND gate is shown in ([122], Section 4.1).

This issue is solved by glitch-resistant masking schemes, such as domain-oriented masking [11] or threshold implementation [10,122], which is further described in Section 6.3.2. Lower overhead of these schemes can be once again achieved by using a composite field S-box computation [123,124].

#### 6.3.1. Pre-Computed Masked Substitution Boxes

Pre-computed masked S-boxes were originally proposed for first-order masked software implementations [9] and later utilized in hardware (FPGA) by using block RAM [14] or more efficient CFGLUT [117] primitives. In the following paragraphs, the concept will be described as used for PRESENT [125] encryption. PRESENT is a lightweight substitution-permutation network-based cipher with a block size of 64 bits and possible key sizes of 80 or 128 bits. Each round consists of a round key addition (XOR), a non-linear substitution layer (4-bit S-boxes applied 16 times in parallel), and a linear permutation layer. After 31 rounds, the 32nd round key is finally added to produce the ciphertext.

Assuming the PRESENT encryption algorithm accepts plain text pt masked by XORing a random mask *m*:

(38) $$state^{\prime }:=pt⊕m,$$

where $state^{\prime }$ is the masked cipher state, three round operations/layers must be taken into account and altered appropriately so that equation

(39) $$state=state^{\prime }⊕m$$

holds, allowing the ciphertext to be obtained using $state^{\prime }$.

The first layer, round key addition, i.e., XOR, is a commutative and associative operation:

(40) $$state^{\prime }⊕rk=\left(state⊕rk\right)⊕m.$$

Therefore, addition of the round key rk does not require any further alteration since the output of the layer is already equal to the valid cipher state masked by *m*.

The last layer, the permutation layer, is a linear transformation *P*, used to permute bits of the cipher state. The output of the layer is therefore equal to the valid cipher state masked by a permuted mask:

(41) $$P\left(state^{\prime }\right)=P\left(state\right)⊕P\left(m\right),$$

which means the mask that would need to be subtracted to obtain the valid cipher state changes to P(m).

The middle layer is a non-linear substitution layer *S*. The validity of the output is assured by altering the substitution look-up table into a masked substitution layer $S^{\prime }$

(42) $$S^{\prime }\left(state^{\prime }\right):=S\left(state^{\prime }⊕m\right)⊕P^{-1}\left(m\right),$$

which realizes the original substitution upon masked input value and outputs the substitution result masked by *m* processed with inverse permutation $P^{-1}$. This approach countermands the mask alteration performed by the Permutation layer, since

(43) $$P\left(state⊕P^{-1}\left(m\right)\right)=P\left(state\right)⊕P\left(P^{-1}\left(m\right)\right).$$

Therefore, $S^{\prime }$ is the only alteration which must be performed for Equation (39) to hold.

In this example, the mask *m* is used through entire encryption, allowing usage of a single precomputed substitution layer. However, special care must be taken when the round state is written to a CMOS register holding the previous round state. Assuming the Hamming distance leakage model, the mask *m* would get subtracted:

(44) HD(x⊕m,y⊕m)=HW(x⊕y⊕m⊕m)=HD(x,y).

One possible solution to this problem is a combination with a register precharge hiding countermeasure [117], where the working register is doubled and the encryption context is interleaved with random data.

#### 6.3.2. Threshold Implementation

Threshold implementation [10,122] is a glitch-resistant masking scheme suitable for both hardware and software implementations [126].

According to the selected masking order *d*, the input is first split into d+1 shares as described in Equation (36). Linear operations during computation are performed on each share independently as described in Equation (37). Each non-linear operation *f* is split into d+1 shared functions $f_{0},\ldots ,f_{d}$ over which the following properties are defined: correctness, non-completeness, and uniformity.

Correctness property assures that the correct result of *f* can be obtained after the computation:

(45) $$\overset{d}{\underset{i=0}{⨁}}f_{i}\left(x_{0},\ldots ,x_{d}\right)=f\left(\overset{d}{\underset{i=0}{⨁}}x_{i}\right).$$

Non-completeness property requires each function $f_{i}$ to be independent of at least one share of each input variable, e.g.,

(46) $$\begin{array}{cc} f_{0}(x_{1},x_{2}, & \ldots ,x_{d}),\\ f_{1}(x_{0},x_{2}, & \ldots ,x_{d}),\\ \; & \ldots \\ f_{d}(x_{0},x_{1}, & \ldots ,x_{d-1}). \end{array}$$

For the masking scheme to protect against higher-order attacks, the property must be extended to the *d*-th order non-completeness [127]: any combination of up to *d* shared functions $f_{i}$ must be independent of at least one share of each input variable.

Similarly, as the inputs $x_{i}$ are uniformly shared, which is assured by generating uniform masks, the uniformity property requires the output of the shared functions $f_{i}$ to be uniformly shared as well. Unlike previous properties which can be explicitly validated, uniformity is typically checked by using an exhaustive enumeration and conditional probability examination. Since uniformity is often hard to achieve directly, remasking with a fresh randomness may be necessary after the non-linear stage [128].

To eliminate the propagation of glitches, assure non-completeness when consecutive non-linear operations are considered, and possibly split a single non-linear operation, pipeline registers must be used between the stages. At least d+1 shares are required to implement a function of algebraic degree *d* (e.g., Rijndael/AES S-box has algebraic degree 7). Splitting the non-linear stage (e.g., decomposing the function or computing the S-box in a composite field) may result in functions of a smaller algebraic degree; therefore, a smaller number of shares and lower overall overhead [123,129].

## 7. Attacks on Protected Implementations

Approaches to attacking protected implementations are presented in this section. Attacks on hiding countermeasures are summarized in Section 7.1 and attacks on masking are explained in Section 7.2.

Most of the presented techniques are typically performed as a pre-processing step before mounting an attack. Moment-based attacks (recall Section 3) on masking can be computed in an online and parallel fashion [130], sparing computing resources. Machine learning-based attacks may even be mounted on protected implementations in a same fashion as when attacking unprotected implementations [42].

### 7.1. Attacks on Hiding

Different approaches were proposed to deal with hiding in time. Simple time shifts can be overcome by using autocorrelation, i.e., a correlation of a signal with a delayed copy of itself. More generally, a pattern near the sensitive operation can be used with matching techniques known from digital signal processing [28] to identify the time shift and align traces appropriately. These methods are also useful when there is no dependable synchronization signal for measurements (trigger).

When the leakage is spread in time in a more chaotic manner, e.g., by clock jitter, a sliding window attack may be used, where a finite number of (consecutive or not) time samples is summed/integrated to a single value [110]. When attacking implementations with a hiding in time countermeasure in place, this attack results in better signal-to-noise ratio. However, because the noise is integrated as well, it is still less efficient than a direct attack on an unprotected implementation [110].

Another example of an attack on hiding in time is the use of elastic alignment attack [131], which utilizes dynamic time warping techniques [132] to create well-aligned traces.

Machine-learning based attacks were shown to successfully break through hiding in time countermeasures, most importantly convolutional neural networks, thanks to their location-scale invariance properties [57].

Similarly, different techniques were proposed for attacking hiding in amplitude countermeasures. Differential logic (such as WDDL) without proper place and route constraints can be successfully attacked by using electromagnetic analysis [133], as the attacker is able to measure leakage from only a small part the chip. Various approaches to filter out excessive noise (such as that created by noise generators) were also proposed [134,135,136,137], e.g., based on wavelet transform.

### 7.2. Attacks on Masking

Consider a moment-based non-profiled attack (e.g., DPA or CPA). With a masking countermeasure in place, the intermediate sensitive variable $f_{k}\left(x_{i}\right)$ is split into *d* shares (recall Equation (36)). Side-channel attack targeting this intermediate value, therefore, must consider *d* mutually independent leakages. Such an attack is then referred to as a higher-order, or *d*-th order, attack [6]. The *d* leakages may manifest themselves at different times, resulting in a multivariate higher-order attack. Similarly, when the leakages manifest at the same time, a univariate higher-order attack is mounted.

The combining function C is used to combine multiple key-independent noisy distributions to produce a single key-dependent distribution, which is then exploited by the attack. Two different combining functions are presented in this subsection: absolute difference combining and product combining. The centralized absolute difference combining between two time samples $o_{x_{i}}\left(t_{1}\right),o_{x_{i}}\left(t_{2}\right)$ with mean values $\mu_{o(t_{1})},\mu_{o(t_{2})}$ is defined as [138]

(47) $$C\left(o_{x_{i}}\left(t_{1}\right),o_{x_{i}}\left(t_{2}\right)\right)=\left|\;\left(o_{x_{i}}\left(t_{1}\right)-\mu_{o(t_{1})}\right)-\left(o_{x_{i}}\left(t_{2}\right)-\mu_{o(t_{2})}\right)\;\right|,$$

and the centralized product combining between arbitrary number of samples is defined as [8]

(48) $$C\left(o_{x_{i}}\left(t_{1}\right),\ldots ,o_{x_{i}}\left(t_{d}\right)\right)=\prod_{k\in \{1,\ldots ,d\}}\left(o_{x_{i}}\left(t_{k}\right)-\mu_{o(t_{k})}\right).$$

The centralization, i.e., subtracting the mean, normalizes the Gaussian noise and minimizes bias during the combining [139]. Optimal leakage function $\hat{L}$ to use with combined leakage is then combining function specific.

Assume attacking a first-order masking scheme, i.e., a second-order attack, using Hamming weight model. Denote the targeted intermediate variable $z=f_{k}\left(x_{i}\right)\in B^{n}$, and let it be split in two shares $s_{0},s_{1}\in B^{n}$:

(49) $$s_{0}=z⊕m,\;\;\;\;s_{1}=m,$$

where $m\in B^{n}$ is a uniform independent mask. Model the observed leakage during processing of each share as

(50) $$O_{0}=\delta_{0}+\mathrm{HW}\left(s_{0}\right)+B_{0},\;\;\;\;O_{1}=\delta_{1}+\mathrm{HW}\left(s_{1}\right)+B_{1},$$

where $\delta_{0},\delta_{1}$ are constant parts of the leakage, and $B_{0},B_{1}∼N\left(0,\sigma \right)$ are zero-centered Gaussian noise. For Hamming distance model, assume usage of $z^{\prime }=z⊕v_{1}^{0}⊕v_{1}^{1}$, $m^{\prime }=m⊕v_{1}^{1}$ instead of z,m, where $v_{1}^{0},v_{1}^{1}$ are previous states of z,m, respectively. The optimal leakage prediction function $\hat{L}$ for the centered absolute difference combining is then [139]

(51) 21−HW(z)HW(z)HW(z)−1⌊HW(z)2⌋,

i.e., a non-affine function of the Hamming weight. The optimal leakage prediction function for the centered product combining is [139]

(52) $$-\frac{1}{2}\mathrm{HW}\left(z\right)+\frac{n^{2}+n}{4}+\frac{n}{2}\left(\delta_{1}+\delta_{2}\right)+\delta_{1}\delta_{2},$$

i.e., a linear function of the Hamming weight. The centered product combining is therefore well-suited for a correlation attack (CPA), where simple HW(z) predictions will show up as a negative correlation. Assuming very noisy observations, the centered product combining function leads to a more efficient attack than the absolute difference combining [139]. Moreover, a higher-order attack using centered product combining can be computed in a one-pass and parallel fashion [130], instead of pre-processing the data.

As mentioned earlier, the higher-order attack can be either univariate or multivariate. Multivariate attack is usually used when attacking masked software implementations [138] and may require a prior points-of-the-interest analysis (recall the template attack in Section 4.1); otherwise, it may become very expensive in terms of both computational power and memory. Univariate attack is suitable when the implementation leaks information about all of the shares in the same time instant [140], which is usually the case for hardware implementations. In such cases, the time sample will be combined with itself, e.g., ${\left(o_{x_{i}}-\mu_{o}\right)}^{2}$ assuming a second-order attack and product combining. Notice that the combining result is equal to the second central moment.

Combining the samples also results in an amplification of the noise [8]. The amount of side-channel information necessary for a successful attack grows exponentially with the masking order. Assuming variance of a single observation is $\sigma^{2}$, variance of *k* combined samples is approximately ${\left(\sigma^{2}\right)}^{k}$, and to distinguish between two distributions with different means and ${\left(\sigma^{2}\right)}^{k}$ variance, approximately ${\left(2\sigma^{2}\right)}^{k}$ samples are necessary [8]. Therefore a sufficient noise level is necessary for masking countermeasures to be secure [141].

Mutual information analysis is, unlike DPA or CPA, “naturally higher-order” in the sense of examining the entire underlying distribution instead of statistical moments [49]. For a multivariate attack, there are different methods of combining multiple time samples: (1) considering them a *d*-dimensional vector, (2) computing multivariate mutual information, or (3) computing total correlation. Multi-dimensional probability density function must then be estimated.

Machine learning-based attacks were also shown capable of exploiting higher-order leakage [42,142] and successfully breaking masked implementations. Compared with CPA, a machine-learning based attack might not require any alterations, and it may be performed on both unprotected and protected implementations with no adjustments [42].

## 8. Leakage Assessment

Leakage assessment methodology examines whether the implementation leaks information. A naïve technique of testing vulnerability against side-channel attacks would be mounting all the known attacks. Leakage-assessment methodologies offer a more general and less computationally and time-demanding approach. Similarly to the non-profiled attacks, the methods presented in this section are typically based on partitioning the measurements and examining their distinguishability. Contrary to the attack scenario, the evaluator in this scenario has full control over the implementation. The described tests can be categorized as either specific or non-specific tests [143,144].

The specific tests [143] typically evaluate measurements of random uniform plain text encryptions with a fixed key. They partition the measurements into two or more groups according to a selected intermediate value and leakage function. For example, assuming single-bit Hamming weight leakage (similarly to DPA in Section 3.1), the measurements are partitioned into two groups according to the value of a bit of S-box output. Considering Rijndael/AES, this intermediate value provides 128 different partitionings (one for each bit of the cipher context). Other options for the intermediate value include round output or XOR of round input and output. Distinguishability of the groups then suggests the possibility of presence of leakage exploitable by targeting the selected intermediate value.

The non-specific tests [143,144] do not target a specific intermediate value. Instead, e.g., distinguishability between two groups containing measurements of an encryption of either random uniform plain text, or of pre-selected fixed plaintext, is tested. Such tests are referred to as random vs. fixed tests. The other choice is a fixed vs. fixed test. In such tests, both groups must be measured in a randomly interleaved fashion during a single evaluation to prevent false results, e.g., due to environmental noise or varying device temperature [144]. Distinguishability of the two groups once again suggests information leakage. Non-specific tests are more sensitive and general than specific tests, and they provide only limited information about the leakage origin.

Various statistical tools can be used to test the distinguishability of the groups. Methodologies based on Welch’s *t*-test and Pearson’s $\chi^{2}$ test are described in Section 8.1 and Section 8.2. A deep learning-based approach is described in Section 8.3.

The measurement setup plays a crucial role in leakage evaluation. Test equipment (e.g., oscilloscope) with sufficient bandwidth, sampling rate and resolution must be used [143], as these and other parameters have a direct impact on potential attack success [30]. A pre-amplifier may be used to ensure the full range of the ADC is utilized.

Relevant pre-processing should also be performed, especially when evaluating secured implementations (see Section 7). The role of evaluator is always creative and non-trivial in the sense that the evaluator should consider all possible techniques the attacker may use to increase the chance of attack success. Results of the presented methods must be interpreted carefully, with possible false positives and false negatives in mind [141].

### 8.1. Welch’s t-Test

A two-tailed Welch’s *t*-test can be used to examine a null hypothesis that two groups’ means are equal, and can be successfully used in leakage assessment [143,144]. The univariate statistic *t* is computed for the two groups, in every sampling point independently:

(53) $$t=\frac{\bar{X_{1}}-\bar{X_{2}}}{\sqrt{\frac{s_{1}^{2}}{N_{1}}+\frac{s_{2}^{2}}{N_{2}}}},$$

where $\bar{X_{1}}$, $\bar{X_{2}}$ are sample means, $s_{1}^{2}$, $s_{2}^{2}$ are sample standard deviations, and $N_{1}$, $N_{2}$ are cardinalities of the first and the second group, respectively. The number of degrees of freedom *v* can be estimated by using

(54) $$v\approx \frac{{\left(\frac{s_{1}^{2}}{N_{1}}+\frac{s_{2}^{2}}{N_{2}}\right)}^{2}}{\frac{{\left(\frac{s_{1}^{2}}{N_{1}}\right)}^{2}}{N_{1}-1}+\frac{{\left(\frac{s_{2}^{2}}{N_{2}}\right)}^{2}}{N_{2}-1}}.$$

Under the null hypothesis, the statistic *t* follows Student’s t-distribution with *v* degrees of freedom. The null hypothesis is rejected according to the distribution and selected significance level α. For sufficiently large *n*, the t-distribution can be satisfactorily approximated by normal distribution. In side-channel analysis, the threshold ±4.5 or ±5 for the t-value is often considered [141,143], which roughly corresponds to significance level $\alpha \leq 10^{-5}$. Rejecting the null hypothesis suggests that the two groups have different means, and therefore an information leakage. Not rejecting the null hypothesis suggests nothing; most importantly, it does not suggest there is no leakage.

The Welch’s *t*-test is a univariate moment-based statistic, similar to statistics used in DPA or CPA attacks (Section 3.1 and Section 3.3). The measurements therefore must be aligned. To evaluate leakage exploitable by higher-order attacks, e.g., when evaluating a higher-order masking scheme, relevant (pre-)processing must be performed [144], similarly to the attacks. This includes a use of either univariate or multivariate combining function as described in Section 7.2.

### 8.2. $\chi^{2}$ Test

Pearson’s $\chi^{2}$ test of independence tests a null hypothesis that two or more variables are independent, and are well-suited for leakage assessment [145]. Unlike the *t*-test, $\chi^{2}$ is a nonparametric test: instead of statistical moments, whole underlying distributions are considered. In this subsection, the univariate test is described first, as in case of the *t*-test, i.e., the test is performed at every sampling point independently.

A two-row (r=2) contingency table *F* is created by using histograms of both groups (assuming aligned histograms, i.e., the same range and width of bins), where the number of columns *c* corresponds to the number of bins. Columns containing only zeros should be eliminated to decrease number of degrees of freedom. Let $F_{i,j}$ be the frequency of each cell, and *N* be the number of all measurements. The expected frequency of each cell $E_{i,j}$ is then computed as

(55) $$E_{i,j}=\frac{\left(\sum_{k=0}^{c-1}F_{i,k}\right)\cdot \left(\sum_{k=0}^{r-1}F_{k,j}\right)}{N},$$

the $\chi^{2}$ statistic *x* as

(56) $$x=\sum_{i=0}^{r-1}\sum_{j=0}^{c-1}\frac{{\left(F_{i,j}-E_{i,j}\right)}^{2}}{E_{i,j}},$$

and the number of degrees of freedom *v* as

(57) v=(c−1)·(r−1).

Under the null hypothesis, the statistic *x* follows $\chi^{2}$ distribution with *v* degrees of freedom. The null hypothesis is rejected according to the distribution and selected significance level α, similarly to Welch’s *t*-test (Section 8.1). Once again, rejection of the null hypothesis suggests information leakage.

Because $\chi^{2}$ is a nonparametric test, univariate higher-order leakage is considered inherently. To extend the test to a multivariate case, either the combining function can be utilized (as in case of the *t*-test), or a multivariate histogram can be used. The $\chi^{2}$ test also enables more than two groups to be used in the test, and it can be also used in an attack scenario similar to the *t*-test in DPA [145].

### 8.3. Deep Learning Leakage Assessment

The distinguishability of the two groups can also be successfully tested by using a deep learning-based classifier [146]. Assuming there is exploitable leakage, the classifier should be able to learn distinctive features of measurements in each group.

First, the measurements get standardized by subtracting the mean value and then dividing it by the standard deviation, at every sampling point independently. Henceforth, for the classifier, the measurements are considered multivariate vectors. The data are split into training and evaluating sets. The leakage assessment only examines leakage in measurements used in the training stage.

Under a *null hypothesis* that the classifier did not recognize and learn any features, the number of its correct guesses on the evaluating set should follow binomial distribution with probability $p=\frac{1}{2}$. The null hypothesis is rejected once again according to the distribution and selected significance level.

Deep learning leakage assessment provides a powerful tool thanks to its multivariate nature, ability to identify distinctive features, and its detection sensitivity, which outperforms both Welch’s *t*-test and $\chi^{2}$ test [146]. It displays similar characteristics as machine learning-based attacks (Section 3.6 and Section 4.2).

## 9. Discussion

In this paper, we provided an insight into the side-channel leakage origin and measurements (Section 2) and we presented both non-profiled (Section 3) and profiled (Section 4) attacks as well as attacks on protected implementations with different countermeasures (Section 7).

The non-profiled attacks remain the most powerful in the sense that the attacker can reveal sensitive information, such as cipher keys, with only a little knowledge about the implementation. Correlation power analysis (CPA) is the most effective and efficient attack, assuming the device under attack leaks side-channel information in a “well-behaved and the most common manner”, i.e., the leakage follows a linear Hamming weight/distance leakage model and the observation channel follows the normal distribution. On the other hand, mutual information analysis (MIA) relaxes all these assumptions and it is capable of revealing sensitive information without further knowledge about the leakage model. Unlike CPA, which exploits statistical moments such as mean or (co)variance, the MIA works with probability density estimates, thus considering all the information available. However, its generality is typically at the cost of efficiency when simpler attacks are possible to mount. Contrary to the CPA, its effectivity and efficiency strongly depend on the probability density estimation approach and its parameters. Considering attacks on protected implementation, the MIA may become more handy compared to the CPA, as it allows for easy multivariate combining and it inherently considers higher-order univariate leakage, thus not being as susceptible to noise amplification. Therefore, in some cases of protected hardware implementations, it may be able to reveal sensitive information much more efficiently than the CPA [141]. Other non-profiled attacks, such as Kolmogorov–Smirnov analysis (KSA), remain mostly of theoretical interest. Namely, the KSA was studied and used to demonstrate a direct relationship between susceptibility to side-channel attacks and differential cryptoanalysis. Most recently, non-profiled machine learning attacks have emerged, allowing the exploitation of the advantages of neural networks as discussed in the next paragraph for profiled attacks.

The profiled attacks require the attacker to have a fully controlled copy of the device under attack at her disposal. The attacker then examines the leakage characteristics and tailors the attack to the specific device. Under this assumption, the profiled attacks become much more effective and efficient than the non-profiled attacks; where hundreds of measurements are necessary for the non-profiled attack to succeed, the profiled attack may reveal sensitive information with as little as a single measurement. The template attack uses multivariate Gaussian distribution to model the leakage and to mount the attack. Recently, many machine learning algorithms have been used to model leakage. Their main advantage, most prominently in the case of convolutional neural networks, is a scale and translation invariance and their ability to attack protected implementations without further adjustments of the attack. The main disadvantage of the machine learning attacks lies currently in their limited explainability.

In the Section 5, we presented different metrics related to side-channel analysis. The most prominent and widely used metrics are the success rate and the closely related guessing entropy, which both demonstrate the practical effectivity and efficiency of the side-channel attack. We further presented more theoretical metrics such as the distinguishing margin and the confusion coefficient, which were used to demonstrate the aforementioned relationship between physical and theoretical attacks on cryptography: the more difficult it is to break a cipher by using differential cryptanalysis, the easier it is to break it by using side-channel analysis.

We also presented countermeasures (Section 6) against side-channel attacks. These are typically divided into two categories: hiding and masking. While hiding aims to conceal the leaking information in noise, masking aims to randomize the working variables by splitting them into multiple variables and thus making the attack significantly more difficult. Attacking the split variable typically requires more sophisticated attacks (including sample combining), which often result in noise amplification and exponential growth of the attack complexity. It is therefore beneficial to use both hiding and masking countermeasures simultaneously, as the additional noise introduced by the hiding countermeasures significantly boosts the security of masking countermeasures. However, most countermeasures come with non-negligible overhead (in time or area or both) and their implementation often comes at high costs. The selection of appropriate countermeasures and their implementation is therefore highly use-case-dependent with criticality and cost in mind.

Lastly, we presented leakage assessment strategies in Section 8. These are used to evaluate protected implementations and their security by both manufacturers and certification laboratories. As mounting all the possible attacks is infeasible, the leakage assessment uses more general approaches such as the non-specific tests to evaluate whether statistically significant side-channel leakage can be detected or not. In the case of the detected leakage, there is still no evidence of its practical exploitability. But even more importantly, when no leakage is detected, this still cannot be used as proof of security. While the leakage assessment provides beneficial insights, a thorough examination of the secured implementation by a skilled engineer is always necessary.

## 10. Conclusions

Side-channel analysis has become a widely recognized threat in the last twenty years. It has evolved from simple attacks such as simple or differential power analysis to a complex research field of its own, having a direct impact even on the semiconductor manufacturing technology itself. Nowadays, devices used in both commercial and government sectors must have appropriate certifications to prove themselves secure against side-channel attacks.

In this survey, we described non-invasive vertical side-channel attacks, including machine learning-based attacks, countermeasures against such attacks, and leakage-assessment methodologies. We provided a taxonomy of both attacks and countermeasures with respect to both their theoretical background and the historical context, and we described the advantages and disadvantages of different approaches. In addition to the matter described in this paper, the side-channel analysis further includes horizontal attacks, invasive attacks, or active attacks such as fault injection, which are out of the scope of this paper. Because the countermeasures against all of these attacks are typically expensive and their effectiveness is being put into question with each newly presented attack, further research in the field of side-channel analysis is essential.

Future work in the field of side-channel analysis most importantly consists of security evaluation of protected implementations and security verification. Moreover, as new algorithms are still being proposed (e.g., post-quantum algorithms, light-weight cryptography), their side-channel security must be evaluated, attack vectors must be identified and proper countermeasures proposed. A general approach to side-channel security remains a great challenge due to a consistent back-and-forth between attackers and security engineers. Due to the general inability to foresee all possible threats and weaknesses, the security (including the side-channel security) will always remain a never-ending process instead of the final product.

## Data Availability

Not applicable.

## Abbreviations

The following abbreviations are used in this manuscript:

**Cryptography and Side-Channel Analysis related**	
AES	Advanced Encryption Standard
CPA	Correlation Power Analysis
DDLA	Differential Deep Learning Analysis
DLLA	Deep Learning Leakage Assessment
DM	Distinguishing Margin
DPA	Differential Power Analysis
GE	Guessing Entropy
HD	Hamming Distance
HW	Hamming weight
ID	Identity
iKSA	Inter-class Kolmogorov-Smirnov Analysis
KSA	Kolmogorov-Smirnov Analysis
MIA	Mutual Information Analysis
PPA	Partitioning Power Analysis
RSA	Rivest–Shamir–Adleman cryptosystem
SR	Success Rate
TA	Template Attack
**Digital Design and Electronics related**	
AC	Alternating Current
ADC	Analog-to-Digital Converter
ARM	Advanced RISC (Reduced Instruction Set Computer) Machines
ASIC	Application-Specific Integrated Circuit
CLK	Clock
CMOS	Complementary Metal–Oxide–Semiconductor
DC	Direct Current
EM	Electromagnetic
FPGA	Field-Programmable Gate Array
GND	Ground
HLS	High-Level Synthesis
MOSFET	Metal–Oxide–Semiconductor Field-Effect Transistor
NMOS	N-channel Metal–Oxide–Silicon Transistor
PMOS	P-channel Metal–Oxide–Silicon Transistor
RAM	Random Access Memory
RTL	Register-Transfer Level
SABL	Sense Amplifier-based Logic
SDDL	Simple Dynamic Differential Logic
SNR	Signal-to-Noise Ratio
VHDL	VHSIC (Very High-Speed Integrated Circuits Program) Hardware Description Language
WDDL	Wave Dynamic Differential Logic
XOR	Exclusive OR
**Miscellaneous**	
CNN	Convolutional Neural Network
CPU	Central Processing Unit
DL	Deep Learning
GPU	Graphics Processing Unit
ML	Machine Learning
MLP	Multilayer Perceptron
NIST	National Institute of Standards and Technology
OpenCL	Open Computing Language
OpenMP	Open Multi-Processing
SVM	Support Vector Machine
